# Variability in State-Dependent Plasticity of Intrinsic Properties during Cell-Autonomous Self-Regulation of Calcium Homeostasis in Hippocampal Model Neurons^1^,^2^,^3^

**DOI:** 10.1523/ENEURO.0053-15.2015

**Published:** 2015-08-31

**Authors:** Sunandha Srikanth, Rishikesh Narayanan

**Affiliations:** 1 Cellular Neurophysiology Laboratory, Molecular Biophysics Unit, Indian Institute of Science, Bangalore 560 012, India; 2 Undergraduate program, Indian Institute of Science, Bangalore 560 012, India

**Keywords:** hippocampus, homeostasis, intrinsic plasticity, ion channels, sharp wave ripples, theta frequency oscillations

## Abstract

How do neurons reconcile the maintenance of calcium homeostasis with perpetual switches in patterns of afferent activity? Here, we assessed state-dependent evolution of calcium homeostasis in a population of hippocampal pyramidal neuron models, through an adaptation of a recent study on stomatogastric ganglion neurons. Calcium homeostasis was set to emerge through cell-autonomous updates to 12 ionic conductances, responding to different types of synaptically driven afferent activity. We first assessed the impact of theta-frequency inputs on the evolution of ionic conductances toward maintenance of calcium homeostasis. Although calcium homeostasis emerged efficaciously across all models in the population, disparate changes in ionic conductances that mediated this emergence resulted in variable plasticity to several intrinsic properties, also manifesting as significant differences in firing responses across models. Assessing the sensitivity of this form of plasticity, we noted that intrinsic neuronal properties and the firing response were sensitive to the target calcium concentration and to the strength and frequency of afferent activity. Next, we studied the evolution of calcium homeostasis when afferent activity was switched, in different temporal sequences, between two behaviorally distinct types of activity: theta-frequency inputs and sharp-wave ripples riding on largely silent periods. We found that the conductance values, intrinsic properties, and firing response of neurons exhibited differential robustness to an intervening switch in the type of afferent activity. These results unveil critical dissociations between different forms of homeostasis, and call for a systematic evaluation of the impact of state-dependent switches in afferent activity on neuronal intrinsic properties during neural coding and homeostasis.

## Significance Statement

A growing body of theoretical and experimental evidence points to neuronal maintenance of calcium homeostasis. The maintenance of such constancy in the face of perpetual switches in behaviorally driven afferent activity is a paradox, and has not been quantitatively assessed. We assessed cell-autonomous calcium homeostasis in a population of hippocampal model neurons subjected to switches in afferent activity. We found that neuronal conductances and intrinsic properties could undergo variable and significant plasticity toward maintenance of calcium homeostasis through a regime of such behavioral state-dependent changes. Our results also reveal that the maintenance of calcium homeostasis does not necessarily translate to the emergence of individual channelostasis or of functional homeostasis (including firing rate), thereby establishing critical dissociations between different forms of homeostasis.

## Introduction

Afferent activity patterns to hippocampal pyramidal neurons manifest well established distinctions that reflect the behavioral state of the animal (Anderson et al., 2007). Whereas rapid eye movement (REM) sleep and exploratory activity are associated with theta-dominant oscillatory (4–10 Hz) inputs, non-REM sleep and nonexploratory activity correspond to sharp-wave ripples (SWRs; ripple frequency, 100–200 Hz) riding on largely silent (inter-SWR frequency, 1–3 Hz) periods (Buzsáki, 1986, 1989, 2002,2006; Buzsáki et al., 1992; Wilson and McNaughton, 1994; Ylinen et al., 1995; Csicsvari et al., 1999; Louie and Wilson, 2001; Tononi and Cirelli, 2006; Montgomery et al., 2008; Mizuseki et al., 2011; Grosmark et al., 2012; English et al., 2014). Additionally, a growing body of theoretical and experimental evidence points to neuronal maintenance of calcium/activity homeostasis, through changes in synaptic and/or intrinsic properties (LeMasson et al., 1993; Siegel et al., 1994; Turrigiano, 1999; Turrigiano and Nelson, 2000, 2004; Prinz et al., 2004; Triesch, 2007; Turrigiano, 2007; Marder, 2011; Honnuraiah and Narayanan, 2013; Marder et al., 2014; O'Leary et al., 2014). How do neurons reconcile the maintenance of calcium homeostasis with perpetual state-dependent switches in afferent activity patterns? Existing literature has explored switches in afferent activity from the perspective of firing rate modulation, synaptic normalization and plasticity, especially during sleep—(Tononi and Cirelli, 2006; Chauvette et al., 2012; Grosmark et al., 2012; Barnes and Wilson, 2014)—and from the perspective of how dendritic nonlinearities endow hippocampal neurons with the ability to adapt to changes in afferent activity (Gasparini and Magee, 2006). However, the question of how neurons implementing calcium homeostasis through changes in ionic conductances react to state-dependent switches in afferent activity has not been addressed. Specifically, under a self-regulating, cell-autonomous schema for calcium homeostasis, are there changes in neuronal firing, conductance values, and intrinsic properties that are consequent to switches in afferent activity?

To address this, we first arrived at a population of 78 experimentally constrained (with 7 different physiological measurements) CA1 pyramidal neuron models involving 12 ion channels, derived from a randomized population of 4000 models built from uniform sampling of 48 different model parameters. Next, we adapted a recent study on cell-autonomous self-evolution of calcium homeostasis in neurons of the crab stomatogastric ganglion (O'Leary et al., 2014) to hippocampal neurons. We ensured that the adapted model included ion channels derived from hippocampal pyramidal neurons, was endowed with detailed calcium-handling mechanisms (including pumps, buffers and the endoplasmic reticulum; Ashhad and Narayanan, 2013) and received different types of afferent activity through AMPA and NMDA receptors. The temporal evolution of messenger RNAs (mRNAs) and conductances corresponding to each of the 12 channels was independently monitored in each of the 78 valid models, with the time courses of mRNA evolution controlled by ionic conductances obtained from the corresponding valid model. Within this modeling framework for cell-autonomous evolution of calcium homeostasis, we tested the impact of switches in afferent activity (between theta oscillations and SWR inputs) on neuronal conductances and intrinsic properties.

Our results suggest that neuronal ion-channel conductances and intrinsic properties could undergo significant plasticity in the process of maintaining calcium homeostasis through a regime of behavioral state-dependent changes in afferent activity. The sign and strength of such intrinsic plasticity were dependent on the specific activity pattern, the temporal sequence of switches, and the specific neuronal model. These results call for a significant reassessment of the impact of state-dependent switches in afferent activity on neuronal and network physiology, especially accounting for potentially adaptive changes in intrinsic properties.

## Materials and Methods

### Neuronal model and ion channels

**Figure 1.**
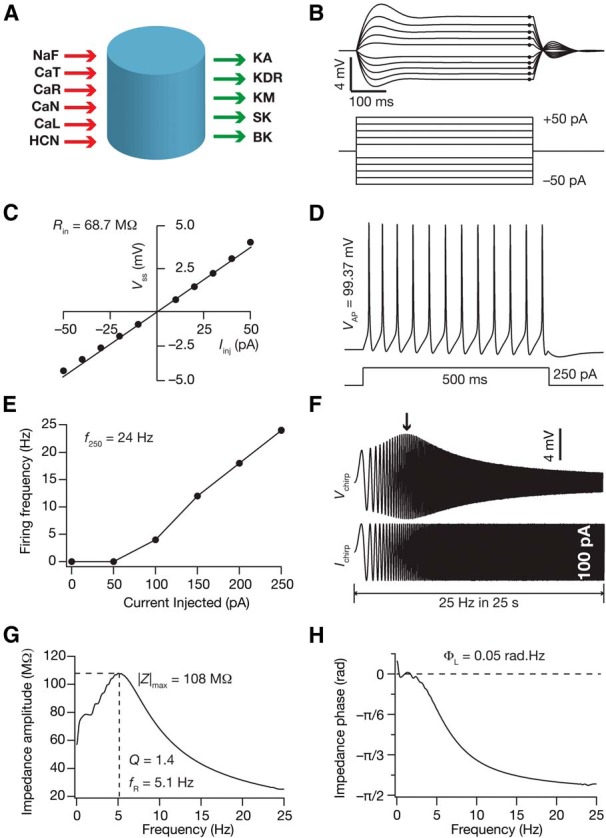
Measurements of intrinsic response dynamics in the base neuronal model. ***A***, The cylindrical model used in this study showing the 11 ion channels inserted. The red arrows denote inward currents and the green arrows denote outward currents. ***B***, Voltage responses (top) of the base neuronal model to current pulses (bottom) ranging from –50 to 50 pA in steps of 10 pA. ***C***, The steady state voltages from (***B***) are plotted against the corresponding current injected. The slope of the resulting *V*–*I* plot was defined as the input resistance, *R*_in_. ***D***, The voltage response of the base neuronal model to a current injection of 250 pA. The amplitude of the last action potential was defined as the action potential (AP) amplitude, *V*_AP_. ***E***, The AP firing frequency (*f*) versus injected current plot showing the frequency of firing with current injections from 0 to 250 pA in steps of 50 pA. The number of APs elicited by the model in response to a 250 pA, 500 ms current pulse was used to compute the firing rate at 250 pA, *f*_250_. ***F***, The model’s voltage response (top) to a chirp current stimulus of peak-to-peak amplitude of 100 pA, with frequency linearly increasing from 0 to 25 Hz in 25 s (bottom). ***G***, The impedance amplitude profile |*Z*(*f*)| derived from traces in ***F***. The frequency at which the impedance amplitude is maximum (|*Z*|_max_) was defined as the resonance frequency, *f*_R_. The strength of resonance, *Q*, was taken as the ratio of |*Z*(*f*_R_)| to |*Z*(0.5)|. ***H***, The impedance phase profile (ϕ(*f*)) with the area under the inductive part of the curve defined as the total inductive phase (Φ_L_).

To study state-dependent and cell-autonomous calcium homeostasis in hippocampal CA1 pyramidal neurons, we used a single compartmental cylindrical model of diameter (*d*) = 100 μm and length (*L*) = 100 μm. Passive properties were set as specific membrane resistance (*R*_m_) = 35 kΩ.cm^2^ and specific membrane capacitance (*C*_m_) = 1 μF/cm^2^. These settings ensured that the passive input resistance (*R*_in_) was ∼111 MΩ and the passive membrane time constant was 35 ms (Narayanan and Johnston, 2007, 2008). The neuronal compartment consisted of 11 conductance-based models for ion channels (Fig. 1*A*) namely, fast sodium (NaF), delayed-rectifier potassium (KDR), *A*-type potassium (KA), *M*-type potassium (KM), *T*-type calcium (CaT), *R*-type calcium (CaR), *N*-type calcium (CaN), *L*-type calcium (CaL), hyperpolarization-activated cyclic nucleotide gated channel (HCN or *h*), small conductance (SK) and big conductance calcium-activated potassium (BK) channels. The channel kinetics for NaF, KDR, and KA were obtained from Hoffman et al. (1997) and Migliore et al. (1999), for CaT from Shah et al. (2011), for KM from Migliore et al. (2006), for CaR and CaL from Magee and Johnston (1995) and Poirazi et al. (2003), CaN and SK from Migliore et al. (1995), for HCN from Magee (1998) and Poolos et al. (2002), and for BK from Moczydlowski and Latorre (1983). The reversal potentials for K^+^ and Na^+^ ions were set as –90 and +55 mV, respectively, and for the HCN channel as –30 mV. Accounting for the leak conductance (*g*_leak_=1/*R*_m_), this configuration meant the presence of 12 ion channels in our model.

### Synaptic receptors

Excitatory synapses containing AMPA and NMDA receptors, modeled using the Goldman–Hodgkin–Katz formulation (Goldman, 1943; Hodgkin and Katz, 1949; Ashhad and Narayanan, 2013) were introduced in the model. Specifically, a canonical synapse consisting of colocalized NMDA receptor (NMDAR) and AMPA receptor (AMPAR) was modeled as in Narayanan and Johnston (2010). The NMDAR current was modeled as a combination of three different types of ionic currents namely Ca^2+^, Na^+^ and K^+^:

(1) $$I_{NMDAR}(v,t)=I_{NMDAR}^{Na}(v,t)+I_{NMDAR}^{K}(v,t)+I_{NMDAR}^{Ca}(v,t),$$

where,

(2) $$I_{NMDAR}^{Na}(v,t)=P_{NMDAR}\;P_{Na}\;\;MgB(v)\frac{vF^{2}}{RT}\left(\frac{{\left[Na\right]}_{i}-{\left[Na\right]}_{o}\;\exp(-\frac{vF}{RT})}{1-\exp(-\frac{vF}{RT})}\right),$$

(3) $$I_{NMDAR}^{K}(v,t)=P_{NMDAR}\;P_{K}\;MgB(v)\frac{vF^{2}}{RT}\left(\frac{{\left[K\right]}_{i}-{\left[K\right]}_{o}\;\exp(-\frac{vF}{RT})}{1-\exp(-\frac{vF}{RT})}\right),$$

(4) $$I_{NMDAR}^{Ca}(v,t)=P_{NMDAR}\;P_{Ca}\;MgB(v)\frac{4vF^{2}}{RT}\left(\frac{{\left[Ca\right]}_{i}-{\left[Ca\right]}_{o}\;\exp(-\frac{2vF}{RT})}{1-\exp(-\frac{2vF}{RT})}\right),$$

where *P*_NMDAR_ defined the maximum permeability of the NMDAR; *P_Ca_*= 10.6, *P_Na_* = 1, *P_K_* = 1 (Mayer and Westbrook, 1987; Canavier, 1999). Extracellular and intracellular concentrations of ions were as follows (in mm): [*Na*]*_i_*=18, [*Na*]*_o_*=140, [*K*]*_i_*=140, [*K*]*_o_*=5, [*Ca*]*_i_*=50 × 10^−6^, [*Ca*]*_o_*=2. These ionic concentrations set the Na^+^ equilibrium potential at +55 mV and K^+^ equilibrium potential at –90 mV. *MgB*(*v*) governed the Mg^2+^ dependence of the NMDAR current (Jahr and Stevens, 1990):

(5) $$MgB(v)={\left(1+\frac{{\left[Mg\right]}_{o}\;\exp(-0.062v)}{3.57}\right)}^{-1},$$

with the default value of [*Mg*]*_o_*set at 2 mm.

Current through the AMPAR was modeled as the sum of currents carried by sodium and potassium ions:

(6) $$I_{AMPAR}(v,t)=I_{AMPAR}^{Na}(v,t)+I_{AMPAR}^{K}(v,t),$$

where,

(7) $$I_{AMPAR}^{Na}(v,t)=P_{AMPAR}\;P_{Na}\;\frac{vF^{2}}{RT}\left(\frac{{\left[Na\right]}_{i}-{\left[Na\right]}_{o}\;\exp(-\frac{vF}{RT})}{1-\exp(-\frac{vF}{RT})}\right),$$

(8) $$I_{AMPAR}^{K}(v,t)=P_{AMPAR}\;\;P_{K}\;\frac{vF^{2}}{RT}\left(\frac{{\left[K\right]}_{i}-{\left[K\right]}_{o}\;\exp(-\frac{vF}{RT})}{1-\exp(-\frac{vF}{RT})}\right),$$

where *P*_AMPAR_ defined the maximum permeability of the AMPAR. *P_Na_* was taken to be equal to *P_K_* (Dingledine et al., 1999). The relationship between AMPAR and NMDAR permeabilities was defined as follows:

(9) $$P_{NMDAR}=NAR\times P_{AMPAR},$$

where NAR represented the NMDAR–AMPAR ratio, with its default value set at 1.5.

### Calcium dynamics

Calcium handling mechanisms to take care of the calcium reactions, radial diffusion, and buffers were adopted from Ashhad and Narayanan (2013). The following partial differential equation to govern the cytosolic calcium dynamics was used (Sneyd et al., 1995; Fink et al., 2000):

(10) $$\frac{d[Ca^{2+}]}{dt}=D_{ca}\nabla^{2}[Ca^{2+}]+\beta (J_{leak}-J_{SERCA})+R_{buf}+J_{VGCC}-J_{pump},$$

where *D*_Ca_ is the diffusion constant for [Ca^2+^] experimentally determined from Allbritton et al. (1992) and Klingauf and Neher (1997); β is the density of leak channels and SERCA pumps on the endoplasmic reticulum (ER) membrane; *J*_VGCC_, *J*_SERCA_, *R*_buf_, *J*_pump_, and *J*_leak_ are the calcium flux due to voltage-gated calcium channels (VGCCs), sarcoendoplasmic reticulum calcium ATPase (SERCA) pumps, static buffers, membrane pumps, and leak channels respectively. Radial diffusion of calcium was taken care of by compartmentalizing the cylinder into four concentric annuli. The calcium concentration on the outermost annulus was considered as the cytosolic calcium, [*Ca^2+^*]_c_ (Carnevale and Hines, 2006; Ashhad and Narayanan, 2013). The calcium influx into the cytosol through the ER leak channels was modeled as follows (Fink et al., 2000; Ashhad and Narayanan, 2013):

(11) $$J_{leak}=L\left(1-\frac{\left[Ca^{2+}\right]}{{\left[Ca^{2+}\right]}_{ER}}\right)\text{mM}/\text{ms},$$

where the leak constant *L* was chosen such that at resting state (–65 mV), there was no net flux of calcium through the leak channels on the ER membrane. The influx of calcium through the VGCCs and NMDARs in our study (*L*-, *T*-, *R*-, and *N*-type calcium channels) was modeled as follows (Poirazi et al., 2003; Ashhad and Narayanan, 2013):

(12) $$J_{VGCC}=-\frac{I_{Ca}\times \pi \times diam}{2\times F}\text{mM}/\text{ms},$$

where *I*_Ca_ represented the calcium current through the VGCCs/NMDARs, *diam* is the diameter of the compartment, and *F* is the Faraday constant. The negative sign indicates the inward nature of *I*_Ca_, and accounts for the positive flux of calcium ions with increase in *I*_Ca_. The uptake of calcium by the SERCA pump was modeled as follows (Fink et al., 2000; Ashhad and Narayanan, 2013):

(13) $$J_{SERCA}=V_{\max}\frac{{\left[Ca^{2+}\right]}^{2}}{{\left[Ca^{2+}\right]}^{2}+K_{p}^{2}}\text{mM}/\text{ms},$$

where *V*_max_ is the average amplitude of uptake by the pump (1 × 10^−4^ mm/ms) and *K*_p_ is the dissociation constant of calcium binding to the pump (0.27 µm). Ca^2+^ extrusion through plasma membrane pumps was regulated by a threshold on the cytosolic calcium ([*Ca^2+^*]_c_). The pumps were inactive below a critical Ca^2+^ concentration, [*Ca^2+^*]_crt_, above which the extrusion rate depended linearly on [*Ca^2+^*]_c_ (Fink et al., 2000):

(14) $$J_{pump}=\{\begin{array}{ccc} \gamma \left({\left[Ca^{2+}\right]}_{c}-{\left[Ca^{2+}\right]}_{crt}\right) & : & {\left[Ca^{2+}\right]}_{c}\geq {\left[Ca^{2+}\right]}_{crt}\\ 0 & : & \text{otherwise} \end{array},$$

where [*Ca* ^2+^]*_crt_* was set at 0.2 µM, and γ (8 µm/s) defines the sensitivity of pump extrusion (Herrington et al., 1996; Fink et al., 2000; Ashhad and Narayanan, 2013). The rate of change in calcium due to the stationary buffers was modeled as follows (Ashhad and Narayanan, 2013):

(15) $$R_{buf}=-k_{on}[Ca^{2+}][B_{buf}]+k_{off}[Ca^{2+}B_{buf}],$$

(16) $$\frac{d[B_{buf}]}{dt}=\frac{d[Ca^{2+}B_{buf}]}{dt}=R_{buf},$$

(17) $$K_{buf}=\frac{k_{off}}{k_{on}},$$

where [*B*_buf_] (=450 μm) and [*Ca* ^2+^*B*_buf_] represented the concentrations of free buffer and calcium bound buffer in the cell. *k*_on_ and *k*_off_ denoted the on and off rate constants for calcium binding to the buffer. Note that Eq. (16) constitutes a pseudo steady-state approximation, considering free buffer and calcium bound buffer to be in equilibrium. The value of *K*_buf_ was set at 10 μm (Klingauf and Neher, 1997; Fink et al., 2000; Ashhad and Narayanan, 2013).

### Measurements

The excitability of the neuronal model was characterized by measuring its firing rate at 250 pA (*f*_250_; Hz), action potential amplitude (*V*_AP_; mV), input resistance (*R*_in_; MΩ) and maximum impedance amplitude (|*Z*|_max_; MΩ). The intrinsic response dynamics of the neuron were characterized by measuring the resonance frequency (*f*_R_; Hz), strength of resonance (*Q*) and total inductive phase (Φ_L_; rad.Hz). These standard measurements (Fig. 1) have been previously used to characterize CA1 pyramidal neurons. Firing rate at 250 pA was taken as twice the number of action potentials fired when a current of 250 pA was injected into the neuron for 500 ms (Fig. 1*D*,*E*). Action potential amplitude was calculated as the difference between the peak voltage of the action potential and the resting membrane voltage (–65 mV). Input resistance was measured by injecting currents of –50 pA to 50 pA, in steps of 10 pA, for 500 ms (Fig. 1*B*), recording the corresponding steady-state voltage deflection from –65 mV and taking the slope of the linear fit to the resulting *V–I* plot (Fig. 1*C*). To quantify the intrinsic response dynamics of the neuron, we injected a current in the form of a sinusoidal chirp stimulus of constant amplitude (50 pA) and a linearly increasing frequency (0–25 Hz in 25 s; Fig. 1*F*). The impedance as a function of frequency (*Z*(*f*)) was obtained by dividing the Fourier transform of the voltage response by the Fourier transform of the injected chirp current. The impedance amplitude profile (Fig. 1*G*) was calculated as the magnitude of this impedance *Z*(*f*) which is given as follows:

(18) |Z(f)|=(Re(Z(f)))2+(Im(Z(f)))2,

where Re(*Z*(*f*)) and Im(*Z*(*f*)) are the real and imaginary parts of the complex valued function *Z*(*f*). The frequency at which |*Z*(*f*)| is maximum is called the resonance frequency, *f*_R_. The impedance amplitude at the resonance frequency, |*Z*(*f*_R_)|, denotes the maximum impedance amplitude, |*Z*|_max_. The strength of resonance, *Q*, was computed as the ratio of the maximum impedance amplitude profile and the impedance amplitude at 0.5 Hz. The impedance phase profile (*ϕ*(*f*); Fig. 1*H*) was calculated as follows:

(19) ϕ(f)=tan−1Im(Z(f))Re(Z(f)).

Total inductive phase was defined as the area under the inductive part of curve *ϕ*(*f*) (Narayanan and Johnston, 2008) which is given by the following:

(20) $$\Phi_{L}(f)=\int_{\phi (f)>0}\phi (f)df.$$

### Global sensitivity analysis

We used the global sensitivity analysis (GSA), a random sampling technique similar to previously used approaches (Bhalla and Bower, 1993; Foster et al., 1993; Goldman et al., 2001; Golowasch et al., 2002; Prinz et al., 2003, 2004; Achard and De Schutter, 2006; Tobin et al., 2006; Reid et al., 2007; Hobbs and Hooper, 2008; Weaver and Wearne, 2008; Taylor et al., 2009; Rathour and Narayanan, 2012b, 2014) to study the effect of the variability in and interactions among the passive and active properties of the neuron on activity-dependent calcium homeostasis. Four thousand models were generated by choosing a unique value for each of the 48 parameters (spanning passive and active properties) from uniform distributions around appropriate base values (Table 1). The base values of parameters were obtained by hand-tuning a base model. As all the 4000 models could not be expected to have biologically realistic measurements, we validated the models by constraining the seven measurements to have values within experimentally determined ranges for CA1 pyramidal neurons (Table 2). Doing so, we found 78 of 4000 (∼2%) neuronal models to be physiologically realistic, and called these as valid models. All further analyses were performed on these 78 valid neuronal models.

**Table 1.** Parameters, their default values in the base model and the range over which random sampling was performed during global sensitivity analysis

	Parameter, unit	Symbol	Default value	Testing range
**Passive parameters**				
1	Specific membrane resistance, kΩ.cm^2^	*R*_m_	35	30 to 40
2	Specific membrane conductance, μF/cm^2^	*C*_m_	1	0.5 to 1.5
**Na channel parameters**				
3	Maximal conductance, S/cm^2^	*Na-g*	0.007	0.005 to 0.01
4	Inactivation time constant, ms	*Na-τ*_h_	2.34	1.87 to 2.81
5	Activation time constant, ms	*Na-τ*_m_	0.163	0.13 to 0.20
6	Slow inactivation time constant, ms	*Na-τ*_s_	106.1	84.88 to 127.32
7	*V*_1/2_ inactivation, mV	*Na-V*_h_	–45	–47 to –43
8	*V*_1/2_ activation, mV	*Na-V*_m_	–30	–32 to –28
9	*V*_1/2_ slow inactivation, mV	*Na-V*_s_	–60	–62 to –58
**KDR channel parameters**				
10	Maximal conductance, S/cm^2^	*DR-g*	0.003	0.001 to 0.005
11	Activation time constant, ms	*DR-τ*_n_	222.9	111.45 to 445.8
12	*V*_1/2_ activation, mV	*DR-V*_n_	13	10 to 15
**KA channel parameters**				
13	Maximal conductance, S/cm^2^	*A-g*	0.008	0.001 to 0.01
14	Inactivation time constant, ms	*A-τ*_l_	2	1 to 4
15	Activation time constant, ms	*A-τ*_n_	0.137	0.086 to 0.43
16	*V*_1/2_ inactivation, mV	*A-V*_l_	–56	–60 to –50
17	*V*_1/2_ activation, mV	*A-V*_n_	11	8 to 15
**CaT channel parameters**				
18	Maximal conductance, mS/cm^2^	*T-g*	0. 1	0.05 to 0. 2
19	Inactivation time constant, ms	*T-τ*_h_	31.02	10.24 to 46.53
20	Activation time constant, ms	*T-τ*_m_	0.858	0.43 to 1.72
21	*V*_1/2_ inactivation, mV	*T-V*_h_	–75	–80 to –70
22	*V*_1/2_ activation, mV	*T-V*_m_	–28	–25 to –15
**HCN channel parameters**				
23	Maximal conductance, mS/cm^2^	*h-g*	0.08	0.005 to 0.05
24	Activation time constant, ms	*h-τ*_l_	28.5	20.52 to 71.25
25	*V*_1/2_ activation, mV	*h-V*_l_	–81	–85 to –70
**CaL channel parameters**				
26	Maximal conductance, μS/cm^2^	*L-g*	100	50 to 200
27	Activation time constant, ms	*L-τ*_m_	0.189	1.8 to 7.2
28	*V*_1/2_ activation, mV	*L-V*_a_	–27.01	–30 to –24
**CaR channel parameters**				
29	Maximal conductance, μS/cm^2^	*R-g*	100	50 to 200
30	Inactivation time constant, ms	*R-τ*_h_	12.7	6.35 to 25.4
31	Activation time constant, ms	*R-τ*_m_	0.221	0.11 to 0.442
32	*V*_1/2_ inactivation, mV	*R-V*_h_	–39	–43 to –35
33	*V*_1/2_ activation, mV	*R-V*_m_	3	–2 to 7
**SK channel parameters**				
34	*Ca*_1/2_ activation, nm	*SK-Ca*	140	110 to 180
35	Maximal conductance, μS/cm^2^	*SK-g*	1	0.5 to 5
36	Activation time constant, ms	*SK-τ*	196.8	98.4 to 393.6
**BK channel parameters**				
37	Maximal conductance, μS/cm^2^	*BK-g*	1	0.5 to 5
38	Slope of Ca activation (mm)	*BK-k*_1_	4.8 × 10^−4^	2.8 × 10^−4^ to 6.8 × 10^−4^
39	*Ca*_1/2_ activation (nm)	*BK-k*_2_	0.13	0.08 to 0.18
40	Activation time constant, ms	*BK-τ*	8.04	4.04 to 16.08
**KM channel parameters**				
41	Maximal conductance, μS/cm^2^	*M-g*	1	0.5 to 5
42	Activation time constant, ms	*M-τ*	6662	3331 to 13323
43	*V*_1/2_ activation, mV	*M-V*	–40	–45 to –35
**CaN channel parameters**				
44	Maximal conductance, μS/cm^2^	*N-g*	100	50 to 200
45	Inactivation time constant, ms	*N-τ*_h_	1555	777.5 to 3110
46	Activation time constant, ms	*N-τ*_m_	0.942	0.471 to 1.884
47	*V*_1/2_ inactivation, mV	*N-V*_h_	39	35 to 44
48	*V*_1/2_ activation, mV	*N-V*_m_	19.88	15 to 24

**Table 2.** Constraints on measurements for declaring a model to be valid under the global sensitivity analysis paradigm

Measurement, unit	Lower bound	Upper bound
*f*_250_, Hz	10	35
*V*_AP_, mV	90	110
*R*_in_, MΩ	50	90
|*Z*|_max_, MΩ	50	110
*f*_R_, Hz	2	5.5
*Q*	1.01	1.5
Φ_L_, rad.Hz	0	0.15

These bounds were extracted from experimental recordings (somatic recordings) presented by Narayanan and Johnston (2007, 2008) and Narayanan et al. (2010).

### Self-regulation of calcium homeostasis

To study activity-dependent self-regulation of calcium homeostasis in a hippocampal pyramidal neuron, we adapted the model introduced by O'Leary et al. (2014) based on the central dogma of molecular biology (Alberts et al., 2007). Specifically, we used a single transcription factor to regulate calcium-dependent transcription of the twelve channels expressed in the model neuron, with different time constants (τ_*i*_) for the messenger RNA (mRNA) evolution associated with each channel (*m*_i_):

(21) $$\tau_{i}{\dot{m}}_{i}={\left[Ca^{2+}\right]}_{tgt}-{\left[Ca^{2+}\right]}_{c},$$

where [*Ca* ^2+^]*_tgt_* (default value was 200 nM) represented the target value of cytosolic calcium concentration ([*Ca* ^2+^]*_c_*) at which homeostasis should be maintained. The evolution of conductances (*g*_i_) of individual channels from their respective mRNA (translation) was governed by:

(22) $$\tau_{g}{\dot{g}}_{i}=m_{i}-g_{i},$$

where $\tau_{g}$ is the time constant for the translational process, and was set to be identically equal to 10 ms for all 12 conductances. We noted, with an additional set of simulations, that changes to the specific value of τ_*g*_ merely altered the time-course toward reaching steady-state, but not the steady-state values of the conductances. For the mRNAs and the conductances to evolve as functions of the integral of error in calcium with reference to the target calcium (Eqs. 21, 22), we randomized the initial values of *m*_i_’s and *g*_i_’s to be very low. The time constants ($\tau_{i}$) for the evolution of mRNAs with reference to each channel were selected from the corresponding conductances of the valid models obtained from the GSA. Specifically, the time constants ($\tau_{i}$) for transcription were set to different values as follows (O'Leary et al., 2014):

(23) $$\frac{\tau_{j}}{\tau_{i}}=\frac{g_{i}^{k}}{g_{j}^{k}}\;\;,$$

where *i* and *j* correspond to the 12 ion channels in our model, and *k* varies from 1 to 78 and corresponds to the number of valid models obtained from the GSA. As Eq. 23 defines $\tau_{i}$ ratios from *g*_i_ ratios, one of the $\tau_{i}$ values needed to be set to obtain the other values. We set the $\tau_{i}$ value associated with the sodium conductance at 10 ms, and computed the other $\tau_{i}$ values from the appropriate conductance ratios as in Eq. 23. We noted, with an additional set of simulations, that changes to the specific value of the $\tau_{i}$ value for the sodium channel altered the time-course toward reaching steady-state, but not the steady-state values of the conductances. The temporal evolution of transcription and translation were independently assessed for each of the 78 valid models by setting the $\tau_{i}$ values to be dependent on the corresponding conductance ratios in each of these models (O'Leary et al., 2014). The other parameters associated with the model (the half-maximal activation voltages, time constants of ion channels and passive properties) were all derived from the respective valid model from where the $\tau_{i}$ values were derived from.

### Assessing state-dependent evolution of calcium homeostasis

In a manner similar to conditions observed under *in vivo* conditions, changes in afferent input to the neuron were presented as changes to synaptic receptors, specifically to AMPAR and NMDAR permeabilities (Eqs. 2–9). We tested the evolution of calcium homeostasis with two different types of afferent activity patterns that correspond to different behavioral states. The first corresponded to inputs received by hippocampal neurons during awake/REM-sleep state, and were modeled using a theta frequency (8 Hz) sinusoidal modulation (Buzsáki et al., 1983; Harvey et al., 2009) of AMPAR/NMDAR permeabilities (Fig. 7*B*). The amplitude of the sinusoidal permeability modulation was such that it was the minimum amplitude required to elicit action potentials in the neuron.

The second type of afferent activity pattern reflected that during non-exploratory/non-REM sleep state (Fig. 7*C*), where the neuron received SWRs riding on largely silent periods (Buzsáki, 1986). The shape of the SWR waveform was derived from English et al. (2014), and SWR amplitude was set such that the response to an SWR input resulted in a membrane voltage change of ∼5–10 mV (English et al., 2014). Specifically, the functional form of SWR inputs was modeled as follows:

(24) $$SWR(t)=\exp\left(-\frac{{\left(t-55\right)}^{2}}{2\times 20\times 20}\right)-0.3\exp\left(-\frac{{\left(t-40\right)}^{2}}{2\times 15\times 15}\right)\sin(2\pi f_{ripple}t/1000),$$

where *t* represented time in milliseconds, and *f*_ripple_ was the ripple frequency set at 150 Hz. Each SWR waveform lasted for around 150 ms, and was set to repeat at 3 Hz (Buzsáki, 1986; English et al., 2014). Afferent activity of the same type was continued until steady state of evolution in conductances (Eq. 22) was achieved (typically around 150 s), at which point measurements of intrinsic properties were noted and a switch in afferent activity was effectuated as necessary. All switches in afferent activity from theta oscillations to SWR inputs were initiated after a reset pulse to –65 mV for 1 s to avoid depolarization-induced block observed in certain neurons.

### Simulation details

All simulations were performed in the NEURON simulation environment (Carnevale and Hines, 2006) at –65 mV and 35° C with an integration time constant of 25 μs. Temperature dependence of channel kinetics was appropriately accounted for with experimentally measured *Q*_10_ values for each channel. The computational complexity of these simulations was enormous, as there were several differential equations associated with the activation and inactivation gates of the 11 ion channels (excluding the leak channel), the differential equation for the voltage and calcium (including calcium diffusion; Eqs. 10–17), and the differential equations governing the evolution of 12 mRNAs and 12 conductances (Eqs. 21–23). The solutions to all these differential equations were computed at every time step (of 25 µs) over a period of 150 s in achieving steady state for one type of afferent activity (a double switch in afferent activity is >3 × 150=450 s, running for several days in terms of simulation time), for each of the 78 valid models. Data analyses were performed using custom-written code with IGOR Pro (Wavemetrics) and statistical analyses were performed with the R Statistical Package (R Core Team, 2014).

## RESULTS

### Generation of a valid model population through global sensitivity analysis

**Figure 2.**
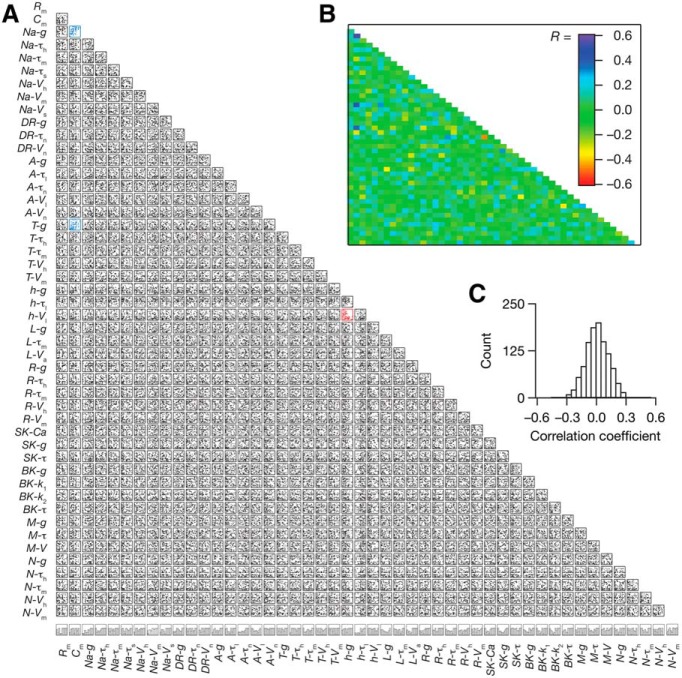
Weak correlations between underlying parameters in valid models that emerged from global sensitivity analysis spanning 48 parameters. ***A***, Pairwise interactions of the 48 parameters of the valid model population consisting of 78 models. Blue scatter plots represent parametric pairs whose correlation coefficient was more than 0.4, whereas red scatter plots indicate pairs with correlation coefficient less than –0.4. Bottom, The normalized histograms of the 48 parameters across the 78 valid models. ***B*,** Color-coded plot denoting the correlation coefficients for the corresponding scatter plots in ***A***. ***C***, Histogram of the 1128 correlation coefficients corresponding to the scatter plots in ***A***.

As a first step in assessing state-dependence of cell-autonomous calcium homeostasis in hippocampal neurons, we generated several biophysically realistic models of CA1 pyramidal neurons using the GSA approach. Specifically, we created a cylindrical base neuronal model (100 × 100 μm) containing 12 ion channels (Leak, NaF, KDR, KA, KM, HCN, CaL, CaT, CaN, CaR, BK, and SK) and AMPA and NMDA receptors (Fig. 1*A*). We hand-tuned the base model such that the seven intrinsic measurements namely, *f*_250_, *V*_AP_, *R*_in_, |*Z*|_max_, *f*_R_, *Q*, and Φ_L_ (Fig. 1*B–H*) were within experimentally determined ranges (Narayanan and Johnston, 2007, 2008; Narayanan et al., 2010). We then uniformly sampled 48 parameters (spanning passive properties and densities/kinetics of channels in the neuron) from a range determined from the corresponding base model values to generate 4000 neurons (Table 1). We obtained seven measurements from each of these 4000 model neurons, and compared the measurements against their experimental counterparts. A model neuron was declared valid if all seven measurements of the model fell within their respective experimental bounds (Table 2). Upon imposing these experimental constraints on measurements from the 4000 models, we found 78 (∼2%) models to be valid. To test whether there were correlations between channel expression profiles and their kinetics, we asked whether there were pairwise correlations between the values of the 48 parameters associated with these 78 valid models. Consistent with previous results on hippocampal neurons (Rathour and Narayanan, 2012a, 2014), we found the parametric values to be weakly correlated (Fig 2*A*) with the range of correlation coefficients ranging from –0.6 to 0.6 (Fig 2*B–C*). Importantly, of the 1128 correlation coefficients, 1125 were in the range of –0.4 to 0.4 suggesting weak pairwise relationships between parameters in the model. As the 78 models were valid models (referred to as GSA models in what follows) for hippocampal pyramidal neuron physiology, we used these for our analysis on state-dependence of intrinsic properties in regulating calcium homeostasis.

### Under theta-frequency afferent activity, calcium-dependent evolution of ionic conductances resulted in variable plasticity of intrinsic properties

**Figure 3.**
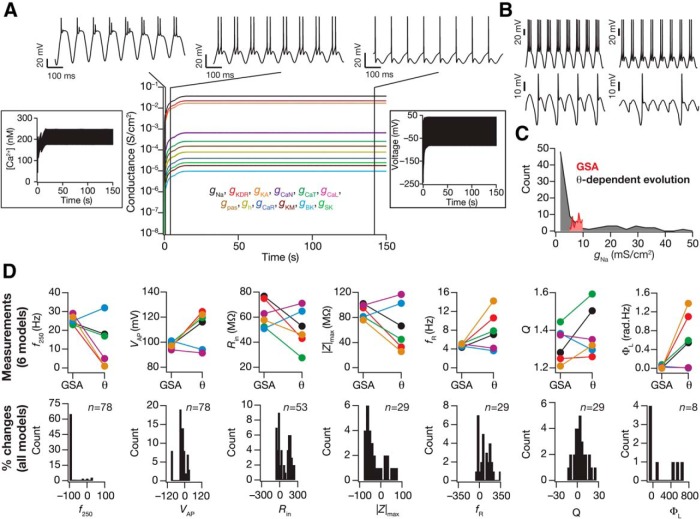
Evolution of ionic conductances and intrinsic measurements through cell-autonomous self-regulation of calcium homeostasis in model neurons receiving theta-frequency inputs. ***A***, Temporal evolution of the internal calcium concentration (left), the 12 ionic conductances in the model (Leak, NaF, KDR, KA, KM, HCN, CaL, CaN, CaR, CaT, BK, SK; middle) and membrane voltage (right) in a model neuron receiving sinusoidal input of 8 Hz. The firing pattern of the neuron is shown at the three different temporal locations (middle). ***B***, Firing pattern of four different model neurons at steady-state of theta-dependent evolution. All traces are for a 1 s period. ***C***, Histogram of *g*_Na_ measured at steady state of evolution with θ-frequency oscillations (black). Also plotted is the histogram of base values of *g*_Na_ obtained from GSA. Histograms are across the 78 valid models. ***D***, Top, Seven intrinsic measurements (*f*_250_, *V*_AP_, *R*_in_, |Z|_max_, *f*_R_, *Q*, Φ_L_) at steady state of theta-dependent evolution (θ) of six different valid models (color-coded) compared with the corresponding baseline GSA values (GSA). Bottom, Histograms of the percentage change in the seven measurements at steady state of theta-dependent evolution from the corresponding baseline GSA values, plotted for all 78 valid models. Percentage change in subthreshold measurements (*R*_in_, |*Z*|_max_, *f*_R_, *Q*, Φ_L_) were computed only for those models that did not fire action potentials in response to the injected stimulus. In addition, models that showed very high percentage changes in Φ_L_ were eliminated. The number of models (*n*) used for each histogram is mentioned in the respective panel.

We set the time constants ($\tau_{i}$) for the evolution of mRNAs with reference to each channel from the corresponding conductances obtained from the valid model (Eq. 23), and implemented the evolution of calcium homeostasis (Eqs. 21, 22) for each of the 78 valid models. The neurons were presented with theta-frequency afferent activity, modeled as an 8 Hz sinusoidal permeability change in synaptic receptors. The mRNAs and conductances were allowed to evolve in time until a steady state was achieved in the cytosolic calcium concentration and the conductance values (Fig. 3*A*). During the initial phase of the evolution process, the voltage response of the model neuron to the sinusoidal input conductance corresponded to large-amplitude oscillations with small action potential amplitudes (Fig. 3*A*). We noted that this was consequent to the initial low values of all conductances, implying a large input resistance leading to large-amplitude voltage oscillations. The small action potential amplitudes, on the other hand, were consequent to the lower values of the spike-generating fast sodium conductance. As the calcium-dependent evolution progressed toward achieving the target calcium value, the voltage response corresponded to large-amplitude action potentials within each theta cycle (Fig. 3*A*).

Although the overall patterns of voltage evolution across the 78 models were similar to the example presented in Fig. 3*A*, there was significant variability in the final steady-state firing behavior of the model. Specifically, there was variability in the number of action potentials fired per cycle and there were models that did not fire at every cycle of the theta, with some of them exhibiting skipping spikes in alternate cycles and others skipping several cycles (Fig. 3*B*). These results imply that the maintenance of calcium homeostasis does not require and does not translate to maintenance of firing rate homeostasis, thereby establishing the dissociation between activity homeostasis and calcium homeostasis.

As neuronal firing properties are dictated by ionic conductances, we asked whether changes in conductance values during theta-dependent evolution was also variable. To answer this, we plotted the histogram of sodium conductances obtained after theta-dependent evolution for the 78 valid models, and found that the variability in firing patterns also reflected in these conductance values (Fig. 3*C*). We plotted the histogram solely for the sodium conductance, and not for all 12 conductances because the changes in all conductances are correlated given that a single transcription factor regulated all conductances (O'Leary et al., 2014; Eqs. 21–23; Fig. 3*A*). These results imply that the maintenance of calcium homeostasis does not require and does not translate to maintenance of individual channels at specific conductance values, thereby establishing the dissociation between individual channelostasis (Rathour and Narayanan, 2014) and calcium homeostasis (O'Leary et al., 2014).

Variability in the percentage change in conductance values would imply that the intrinsic response properties of the neuron should have undergone variable plasticity during the evolution of calcium-dependent homeostasis. Therefore, we calculated the seven intrinsic measurements at steady state of the θ-dependent evolution, and compared these measurements with their corresponding GSA values for each of the 78 valid models. We found that θ-dependent evolution introduced variable plasticity in each of these measurements, with significant variability in the strength and sign of these changes (Fig. 3*D*). Importantly, although the conductance values obtained from GSA were those that satisfied experimental bounds (Table 2), several measurement values at steady state after θ-dependent evolution were not valid with reference to these experimental bounds (Fig. 3*D*). Together these results revealed that plasticity in neuronal intrinsic properties and in conductances could exhibit significant variability when ionic conductances were allowed to evolve toward achieving calcium homeostasis with θ-oscillations as afferent inputs. These results imply that the mere maintenance of calcium homeostasis does not require and does not translate to maintenance of intrinsic measurements within a “valid” range, thereby establishing the dissociation between functional homeostasis and calcium homeostasis.

### Sensitivity of intrinsic plasticity driven by calcium homeostasis to target calcium concentration and to the strength and frequency of afferent activity

**Figure 4.**
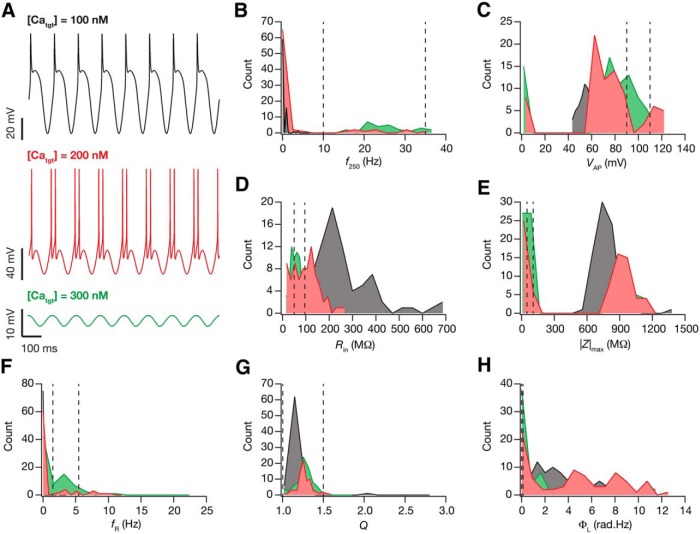
The target value of internal calcium concentration critically regulated changes in intrinsic response properties during cell-autonomous self-regulation of calcium homeostasis. ***A***, The steady-state voltage response after theta-dependent evolution plotted for three different target calcium levels (black, 100 nm; red, 200 nm; green, 300 nm). ***B–H***, Histograms of the steady-state measurement values (*f*_250_, ***B***; *V*_AP_, ***C***; *R*_in_, ***D***; |Z|_max_, ***E***; *f*_R_, ***F***; *Q*, ***G***; Φ_L_, ***H***) for the 78 valid models, obtained after theta-dependent evolution with different target calcium levels. The dashed lines in *B–H* represent the lower and upper bounds for the corresponding measurement (in that order) in the GSA model validation procedure (Table 2).

What was the impact of changing the target calcium concentration, [*Ca* ^2+^]_tgt_ (from the default value of 200 nM), on the temporal evolution of conductances and consequent plasticity in intrinsic properties? To address this, we repeated the θ-dependent evolution experiments (Fig. 3) with two other values for [*Ca* ^2+^]_tgt_, set at 100 and 300 nm. We observed neuronal firing at steady state (150 s) of θ-dependent evolution and found that the neuronal model fired more action potentials per θ-cycle upon increase in [*Ca* ^2+^]_tgt_. However, at a higher target value (300 nM), the neuron entered into depolarization-induced block with the membrane potential hovering at suprathreshold voltage-levels (Fig. 4*A*). Turning to steady-state values of intrinsic properties after θ-dependent evolution, we noted that variable plasticity in all seven intrinsic measurements was observed across all three values of [*Ca* ^2+^]_tgt_, with no qualitative differences observed in measurement variability with different values of [*Ca* ^2+^]_tgt_ (Fig. 4*B–H*).

**Figure 5.**
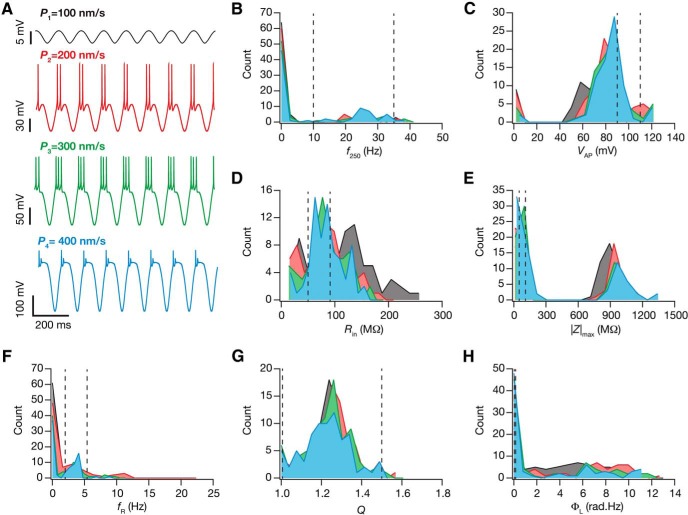
The strength of afferent theta inputs critically regulated changes in intrinsic response properties during cell-autonomous self-regulation of calcium homeostasis. ***A***, The steady state voltage response after theta-dependent evolution, for four different amplitudes of the 8 Hz input sinusoid (peak-to-peak value of sinusoidal permeability: black, 100 nm/s; red, 200 nm/s; green, 300 nm/s; blue, 400 nm/s). Note that at high values of sinusoidal amplitudes (e.g., 400 nm/s) the deflections are large along the hyperpolarized direction because of the large driving force for AMPA/NMDA receptors that mediate the sinusoidal oscillations. Along the depolarized direction, an action potential was elicited once the membrane potential crossed threshold, and the amplitude of the action potential did not cross the sodium reversal potential of +55 mV. ***B–H***, Histograms of the steady state measurement values (*f*_250_, ***B***; *V*_AP_, ***C***; *R*_in_, ***D***; |Z|_max_, ***E***; *f*_R_, ***F***; *Q*, ***G***; Φ_L_, ***H***) for the 78 valid models, obtained after theta-dependent evolution with different sinusoidal amplitudes. The dashed lines in *B–H* represent the lower and upper bounds for the corresponding measurement (in that order) in the GSA model validation procedure (Table 2).

We next assessed the impact of afferent activity strength on θ-dependent evolution by altering the peak-to-peak amplitude of the sinusoidal modulation in receptor permeability, with the sinusoidal frequency fixed at 8 Hz (Fig. 5). Whereas the neuron did not fire action potentials for lower strengths of afferent input (Fig. 5*A*; permeability value *P*_1_), at very high values of input permeability neurons entered into depolarization-induced block with average membrane potential around –30 mV (Fig. 5*A*; permeability value *P*_4_). In the mid-range between these two extremes, the number of action potentials fired per cycle increased with increase in afferent input strength. Assessing steady-state values of intrinsic properties after θ-dependent evolution, we noted that variable plasticity in all seven intrinsic measurements was observed across all tested values of input strength, with no qualitative differences observed in the measurement variability with different values of the sinusoidal peak-to-peak amplitude (Fig. 5*B–H*).

**Figure 6.**
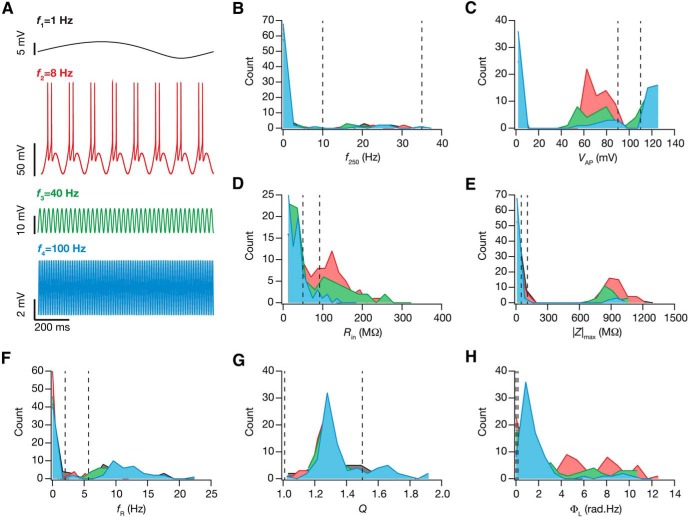
The frequency of afferent inputs critically regulated changes in intrinsic response properties during cell-autonomous self-regulation of calcium homeostasis. ***A***, The steady state voltage response for 4 different sinusoidal frequencies (delta, 1 Hz, black; theta, 8 Hz, red; slow gamma, 40 Hz, green; fast gamma, 100 Hz, blue). ***B–H***, Histograms of the steady state measurement values (*f*_250_, ***B***; *V*_AP_, ***C***; *R*_in_, ***D***; |Z|_max_, ***E***; *f*_R_, ***F***; *Q*, ***G***; Φ_L_, ***H***) for the 78 neurons for different sinusoidal frequencies appropriately color-coded. The dashed lines in *B–H* represent the lower and upper bounds for the corresponding measurement (in that order) in the GSA model validation procedure (Table 2).

Finally, to understand the impact of afferent activity beyond the theta-frequency range on the evolution of calcium homeostasis and intrinsic properties, we picked samples from different frequency bands (delta: 1 Hz, theta: 8 Hz, slow gamma: 40 Hz, and fast gamma: 100 Hz) and repeated our simulations (until steady-state was achieved) with these afferent input frequencies. Neuronal response reflected theta-frequency band-pass structure of hippocampal pyramidal neurons, eliciting maximal firing response at the theta range with reduced response with little or no firing at other frequency bands (Fig. 6*A*). Although the measurements at steady state of calcium-dependent evolution did exhibit significant variability across the 78 models, their values did not have any specific dependence on the frequency of the input (Fig. 6*B*–*H*). For all further analyses, the default input frequency was 8 Hz to take into account the hippocampal theta rhythms, as mentioned above. Together, although neuronal firing pattern was heavily dependent on target calcium values and the strength/frequency of afferent inputs, intrinsic properties measured across all parametric combinations exhibited significant variability in the plasticity consequent to calcium-dependent evolution of conductances.

### Neuronal conductances exhibited differential robustness to an intervening switch in the type of afferent activity

**Figure 7.**
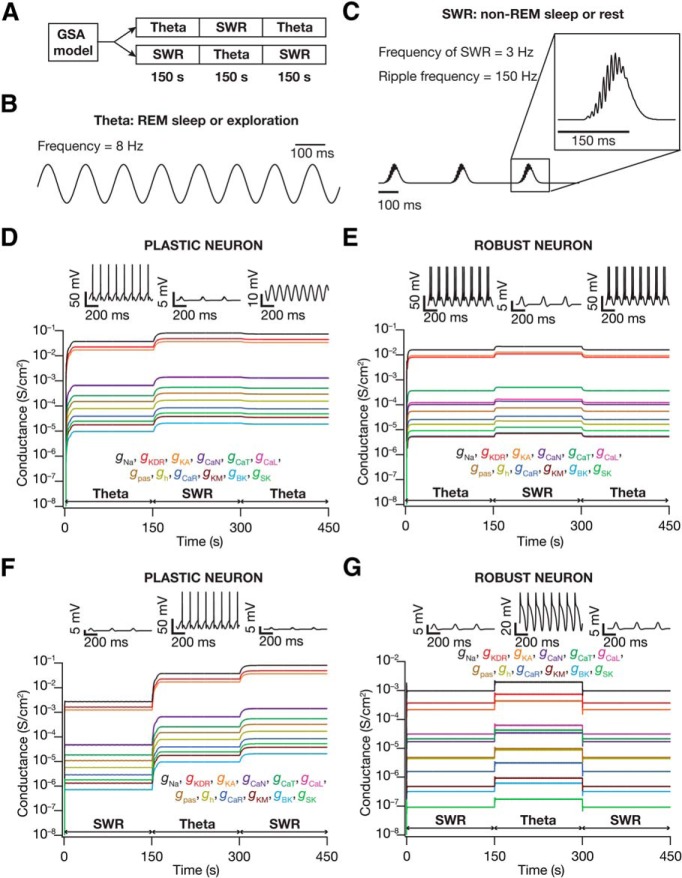
Switch in afferent activity between θ oscillations and SWRs triggered significant changes in ionic conductances during cell-autonomous self-regulation of calcium homeostasis. ***A***, Experimental design for assessing state-dependence of ionic conductances during cell-autonomous self-regulation of calcium homeostasis. For each valid neuronal model obtained from GSA, ionic conductances were allowed to evolve toward achieving calcium homeostasis when afferent inputs were θ oscillations (or SWRs). At steady state of this evolution (150 s), inputs were switched to SWR (or θ oscillations). When this evolution reached steady state (150 s from the first switch), the input was switched back to θ oscillations (or SWR). ***B***, Input received by a neuron during awake/REM sleep state was modeled as theta-frequency oscillations (8 Hz) injected as AMPAR and NMDAR permeabilities. ***C***, Input received by a neuron during non-exploratory/non-REM sleep state was modeled as SWR inputs (inset, ripple frequency *f*_ripple_ was 150 Hz; Eq. 24), repeating at a frequency of 3 Hz. These inputs were injected as AMPAR and NMDAR permeabilities into the neuronal model. ***D***, ***E***, Temporal evolution of the 12 ionic conductances in two different model neurons, where afferent activity switched from θ oscillations to SWR and back to θ oscillations. The calcium concentration (target [Ca]: 200 nM) and the conductances were allowed to reach steady state before either of the switches. The firing patterns of the neuron at the three steady states are also shown. ***F, G***, Same as ***D***, ***E***, but with afferent activity switching from SWR to θ oscillations and back to SWR.

To study the state dependence of such a calcium homeostatic mechanism, we used experimentally well established differences in afferent activity to hippocampal neurons during different behavioral states and during different modules of the sleep cycle (Buzsáki, 2006; Mizuseki et al., 2011; Grosmark et al., 2012). Specifically, during REM sleep or exploratory behavior, afferent activity to the hippocampal CA1 neuron is dominated by oscillations in the theta-frequency (Fig. 7*B*), whereas during non-REM or non-exploratory behavior, the neuron predominantly receives SWRs riding on largely silent background (Fig. 7*C*). In this context, to study state dependence of the autonomous self-regulating calcium homeostasis mechanism, we switched the afferent activity to the neuron between θ-frequency oscillations and SWR activity. We analyzed two different temporal sequences of activity: theta–SWR–theta and SWR–theta–SWR sequences (Fig. 7*A*), with each afferent state of activity lasting for 150 s (to achieve steady-state conductance values with each phase of afferent activity).

Given the formulation of the dynamical evolution, the calcium levels expectedly converged to the target value of calcium, with transient changes occurring during the period that followed the switches. This maintenance of calcium homeostasis was effectuated by alterations in ionic conductances, which accommodated for the switch in afferent activity patterns (Fig. 7*D–F*). Although the extent of plasticity in ionic conductances was a continuum (see below), we classified neurons into two distinct classes based on the amount of plasticity in ionic conductances. The first corresponded to neurons that underwent significant plasticity in their conductance values upon receiving the same type of afferent activity after an intervening period of switch to a different activity pattern (plastic neurons, ∼80%; Fig. 7*D*,*F*). A second class of neurons restored their conductance values, exhibiting similar values upon receiving the same type of afferent activity after an intervening period of switch to a different activity pattern (robust neurons, 20%; Fig. 7*E*,*G*). Concurrently, neuronal response (firing) patterns with identical afferent activity were similar in robust neurons (Fig. 7*E*,*G*), but were significantly different in plastic neurons (Fig. 7*D*).

**Figure 8.**
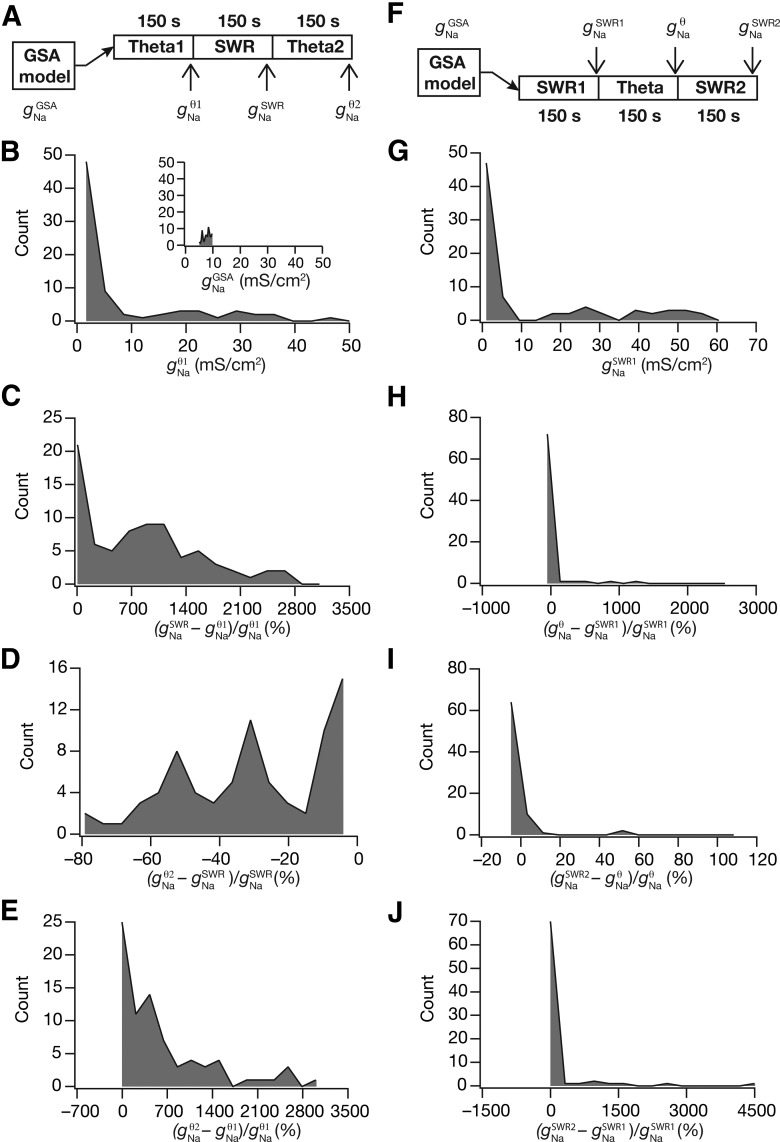
Across different model neurons, switch in afferent activity between θ oscillations and SWRs triggered variable changes in ionic conductances during cell-autonomous self-regulation of calcium homeostasis. ***A***, Schematic showing the temporal sequence of the experiment, along with notations for the temporal locations at which steady-state values of the sodium conductance (*g*_Na_) were measured in the course of their calcium-dependent evolution. Note that this schematic represents a theta–SWR–theta temporal sequence in afferent activity, and the notations here hold for ***B–E***. All histograms depict statistics across the 78 valid models obtained after GSA. ***B***, Histogram of *g*_Na_ values at the steady state of the evolution with θ-frequency oscillations as afferent inputs. Inset, Histogram of base values of *g*_Na_ obtained from GSA. ***C*,** Histogram of percentage changes in *g*_Na_ measured at steady state of evolution with SWR inputs (after θ_1_–SWR), computed with reference to the steady state value after evolution with θ-frequency oscillations (θ_1_). ***D*,** Histogram of percentage changes in *g*_Na_ measured at steady state of evolution with θ-frequency oscillations (after θ_1_–SWR–θ_2_), computed with reference to the steady state value after evolution with SWR inputs (after θ_1_–SWR). ***E*,** Histogram of percentage changes in *g*_Na_ measured at steady state of evolution with θ-frequency oscillations (after θ_1_–SWR–θ_2_), computed with reference to *g*_Na_ measured at steady state of evolution after θ_1_. ***F–J*,** Same as ***A–E***, but for a SWR–theta–SWR temporal sequence in afferent activity, with notations for conductance values shown in ***F***.

We assessed the percentage changes in sodium conductance after each switch in both sequences (across all 78 model neurons), and found that the changes in conductances introduced by the switches were significantly variable (Fig. 8*B–E* for the theta–SWR–theta sequence and G*–J* for the SWR–theta–SWR sequence). We plotted the histogram solely for the sodium conductance, but not for all 12 conductances because the changes in all conductances are correlated given that a single transcription factor regulated all conductances. However, upon reverting back to the same type of afferent activity, a significant percentage of neurons were robust (with reference to conductance values, firing patterns and intrinsic properties) to the intervening switch to another type of afferent activity (Fig. 8*E*,*J*). The robustness of the neuron to an intermediate activity switch, however, was dependent on the specific temporal sequence of activity switch. Specifically, the percentage of neurons robust to an intervening period of activity switch was lower for the theta–SWR–theta (∼20%; Fig. 8*E*) compared with the percentage for SWR–theta–SWR sequence (∼90%; Fig. 8*J*). These results also reveal that the maintenance of calcium homeostasis does not necessarily require or translate to maintenance of individual conductances at specific values (O'Leary et al., 2014), and that significant plasticity in ionic conductances need not necessarily translate to significant changes in afferent-driven firing activity (Fig. 7*F*), thereby revealing a significant dissociation between individual channelostasis, activity/functional homeostasis and calcium homeostasis.

### Neuronal intrinsic properties exhibited differential robustness to an intervening switch in the type of afferent activity

**Figure 9.**
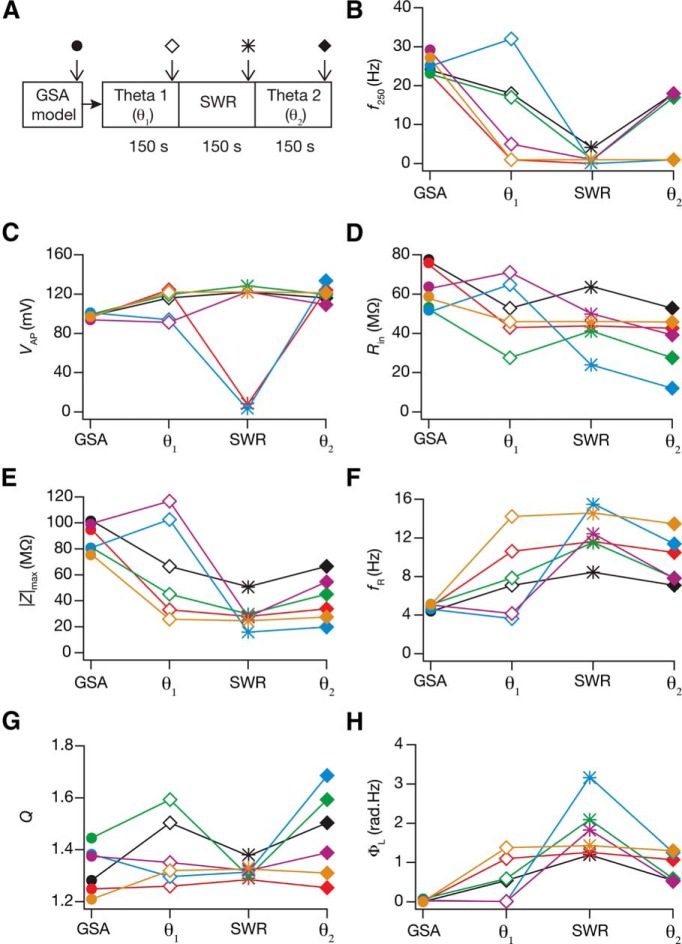
A Theta–SWR–theta switch in afferent activity introduced significant changes to neuronal intrinsic response properties during cell-autonomous self-regulation of calcium homeostasis. ***A***, Experimental design representing a theta–SWR–theta temporal sequence in afferent activity for assessing state-dependence of intrinsic response properties during cell-autonomous self-regulation of calcium homeostasis. The arrows represent time points at which intrinsic measurements were computed, and also associated with different symbols used in ***B****–****H***. ***B–H***, Intrinsic measurements (*f*_250_, ***B***; *V*_AP_, ***C***; *R*_in_, ***D***; |Z|_max_, ***E***; *f*_R_, ***F***; *Q*, ***G***; Φ_L_, ***H***) for six example neurons (different colors) computed for the base valid model (GSA), and at different steady-state time points corresponding to different activity patterns (***A***).

**Figure 10.**
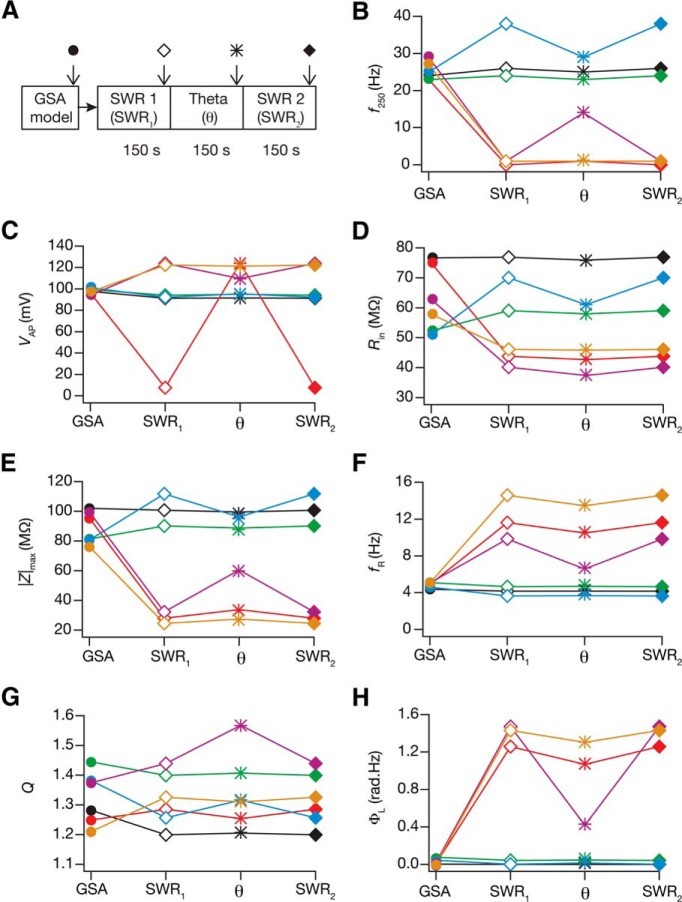
An SWR–theta–SWR switch in afferent activity introduced significant changes to neuronal intrinsic response properties during cell-autonomous self-regulation of calcium homeostasis. ***A***, Experimental design, representing a SWR–theta–SWR temporal sequence in afferent activity, for assessing state-dependence of intrinsic response properties during cell-autonomous self-regulation of calcium homeostasis. The arrows represent time points at which intrinsic measurements were computed, and also associated with different symbols used in ***B****–****H***. ***B–H***, Intrinsic measurements (*f*_250_, ***B***; *V*_AP_, ***C***; *R*_in_, ***D***; |Z|_max_, ***E***; *f*_R_, ***F***; *Q*, ***G***; Φ_L_, ***H***) for six example neurons (different colors) computed for the base valid model (GSA), and at different steady-state time points corresponding to different activity patterns (***A***).

**Figure 11.**
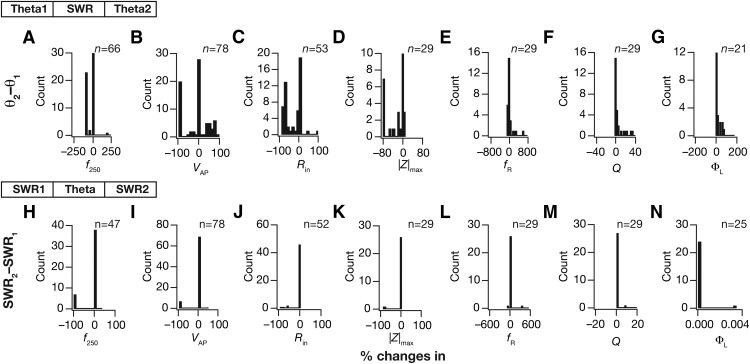
Across different model neurons, switch in afferent activity between θ oscillations and SWRs triggered variable changes in neuronal intrinsic response properties during cell-autonomous self-regulation of calcium homeostasis. ***A–G***, θ_1_–SWR–θ_2_ constitutes the temporal sequence of afferent activity, with notations shown in Figure 9*A*. Histogram of percentage changes in the seven intrinsic response properties (*f*_250_, ***A***; *V*_AP_, ***B***; *R*_in_, ***C***; |Z|_max_, ***D***; *f*_R_, ***E***; *Q*, ***F***; Φ_L_, ***G***) measured at steady state of evolution with θ-frequency oscillations (after θ_1_–SWR–θ_2_), computed with reference to the corresponding base value of the intrinsic property measured at steady state of evolution after θ_1_. Percentage changes in subthreshold measurements (*R*_in_, |*Z*|_max_, *f*_R_, *Q*, Φ_L_) were computed only for those models that did not fire action potentials in response to the injected stimulus. In addition, models that showed very high percentage changes in Φ_L_ were eliminated. The number of models (*n*) used for each histogram is mentioned in the respective panel. ***H–N***, Same as ***A–G***, but for a SWR_1_–theta–SWR_2_ temporal sequence of afferent activity, with notations shown in Figure 10*A*.

Finally, we asked how state-dependent evolution of calcium homeostasis altered neuronal intrinsic properties. To do this, we measured seven intrinsic properties (Fig. 1) at steady states of temporal evolution with activity pattern (theta or SWR) with the theta–SWR–theta (Fig. 9) and the SWR–theta–SWR (Fig. 10) sequences. Analysis of the evolution of intrinsic properties with switch in afferent activity revealed several important observations. First, intrinsic properties underwent significant plasticity as a consequence of these switches in types of afferent activity, and the sign and strength of this plasticity varied across different model neurons. Second, in the process of such activity-dependent evolution, neuronal intrinsic properties did not necessarily fall into their established experimental bounds (Figs. 9, 10; Table 2). These results reveal a significant dissociation between functional and calcium homeostasis. Third, when neurons were presented with identical (the initial pattern preceding the switch) activity patterns after an intervening period of switch in activity type, there were models whose intrinsic measurements did not restore to values before the intervening period. However, a significant proportion of neurons were robust to the intervening period of activity switch, where their intrinsic properties were restored when the activity pattern was switched back (Fig. 11). Similar to our observation with conductances, we noted that the percentage of neurons robust (in terms of changes in measurements) to an intervening period of activity switch was lower for the theta–SWR–theta (Fig. 11*A–G*) compared with the percentage for SWR–theta–SWR sequence (Fig. 11*H–N*). We also noted that neurons fired significantly higher during the theta periods and lesser during SWR periods, which was partly due to overall reduction in intrinsic excitability of the neurons during SWR period (Figs. 7–11).

Together, our results suggest that neuronal conductances and intrinsic properties could undergo significant plasticity toward maintenance of calcium homeostasis in the face of behavioral-state dependent changes in afferent activity. Although a significant population of neurons exhibited robustness to an intervening switch in the type of afferent activity, there were neurons that manifested significantly distinct intrinsic properties upon restoration of the type of afferent activity after the intervening switch. These results suggest that neuronal conductances and intrinsic properties exhibited differential robustness to an intervening switch in the type of afferent activity, apart from demonstrating significant dissociations between functional/channel/firing-rate and calcium homeostasis.

## DISCUSSION

The key conclusion of this study is that neuronal ionic conductances and intrinsic properties could undergo significant plasticity in the process of maintaining calcium homeostasis through a regime of behavioral state-dependent changes in afferent activity. This form of intrinsic plasticity driven by calcium homeostasis was sensitive to the specific value of target calcium and the strength and frequency of afferent activity. We assessed the impact of behavioral state-dependence of afferent activity on calcium homeostasis by switching afferent activity pattern between theta-frequency oscillations (REM sleep/exploratory behavior) and SWR activity (non-REM sleep/non-exploratory behavior). Switches in activity patterns resulted in variable plasticity in ionic conductances and neuronal measurements, with the sign and strength of plasticity dependent on the specific type of activity pattern and on the temporal sequence of switch. Additionally, our analysis with temporal sequences of activity switch revealed the presence of two classes of neurons. One that showed significant plasticity in conductance values and in intrinsic properties when presented with identical activity patterns after an intervening period with a different activity pattern, and a second that restored its conductance values and intrinsic properties after the intervening period. The percentage of robust versus plastic neurons was variable in a manner that was critically dependent on the specific sequence of switch in activity. Finally, our results also reveal that the maintenance of calcium homeostasis does not necessarily translate to the emergence of individual channelostasis or of functional homeostasis (across physiological measurements, including firing rate), thereby establishing critical dissociations between these forms of homeostasis. In what follows, we present certain physiological implications for our conclusions, along with detailed analyses on model assumptions and future directions with testable predictions.

### Behavioral state-dependent changes in neuronal intrinsic properties

Afferent activity patterns in hippocampal pyramidal neurons exhibit well-established distinctions that reflect the behavioral state of the animal (Anderson et al., 2007). The literature on behavioral state-dependent changes in neuronal activity has largely focused on postulates that involve synaptic normalization and synaptic plasticity during activity switches (Tononi and Cirelli, 2006; Chauvette et al., 2012; Grosmark et al., 2012; Barnes and Wilson, 2014), or on how dendritic nonlinearities could differentially process different forms of activity patterns (Gasparini and Magee, 2006). Our results point to a novel form of behavioral state-dependent plasticity in neuronal intrinsic properties, emerging as a direct consequence of the requirement to maintain calcium homeostasis in the face of changes in afferent activity. In this context, it is important that future studies on behavioral-state dependence of neuronal physiology also consider the role of neuronal intrinsic properties and ionic conductances toward changes in overall firing rate (Tononi and Cirelli, 2006; Grosmark et al., 2012), without limiting the analysis to synaptic changes and neuromodulatory influences (LeMasson et al., 1993; Siegel et al., 1994; Turrigiano, 1999; Turrigiano and Nelson, 2000, 2004; Prinz et al., 2004; Triesch, 2007; Turrigiano, 2007; Marder, 2011; Honnuraiah and Narayanan, 2013; Marder et al., 2014; O'Leary et al., 2014). Additionally, although it is established that the propensity of different activity patterns is higher during specific behavioral states, a constant afferent pattern used in the model is physiologically infeasible under *in vivo* conditions. Future experiments should therefore explore the relationships between time constants for channel plasticity under *in vivo* conditions and the temporal extent of specific afferent activity patterns. Although *in vitro* experiments have shown plasticity in several ion channels to occur in a matter of minutes and have demonstrated that changes in ion channels and intrinsic properties can be concurrent with synaptic changes (Frick et al., 2004; Fan et al., 2005; Kim et al., 2007; Narayanan and Johnston, 2007; Lin et al., 2008; Losonczy et al., 2008; Narayanan and Johnston, 2008; Rosenkranz et al., 2009; Narayanan et al., 2010; Remy et al., 2010; Shah et al., 2010), the temporal aspects of activity-dependent plasticity in ion channels under *in vivo* conditions needs to be explored in detail, in a manner that accounts for channelostasis individually and collectively (Hanus and Schuman, 2013; Rathour and Narayanan, 2014; Anirudhan and Narayanan, 2015). Additionally, such experiments could test for variability in such state-dependent intrinsic plasticity predicted by our model, apart from addressing the impact of such variability on neurophysiology and behavior.

As a specific instance, the existence of plasticity in intrinsic properties (in addition to synaptic plasticity) would expand the putative mechanisms that could be involved in memory consolidation, a postulated function for sleep rhythms (Siegel, 2001; Stickgold et al., 2002; Walker and Stickgold, 2004; Stickgold, 2005; Marshall and Born, 2007). As activity-dependent plasticity in neuronal intrinsic properties is well established (Zhang and Linden, 2003; Kim and Linden, 2007; Johnston and Narayanan, 2008; Shah et al., 2010; Narayanan and Johnston, 2012), the exploration of the postulate that memory consolidation is effectuated through intrinsic plasticity (in conjunction with synaptic changes) is a critical prediction that needs rigorous experimental evaluation. Given this postulate where the possibility of intrinsic changes exists, interpretation of observations from experiments that involve replay or disruption of specific activity pattern (Girardeau et al., 2009; Ego-Stengel and Wilson, 2010; Jadhav et al., 2012; Barnes and Wilson, 2014) should also account for changes in neuronal intrinsic properties that might have been brought about by the specific activity pattern or lack thereof.

### Would behavioral state-dependent intrinsic plasticity in a neuronal compartment be dependent on its somatodendritic location?

Although the macroscopic activity patterns recorded in the hippocampus show theta-SWR switches during different behavioral states, it is evident that there are subtle, yet significant, differences in afferent activity at different somatodendritic locations along a hippocampal pyramidal neuron (Colgin et al., 2009; Colgin and Moser, 2010; Bieri et al., 2014; Schomburg et al., 2014). Additionally, there are well-established differences in localization of different ion channels (Johnston et al., 1996; Magee, 2000; Migliore and Shepherd, 2002; Johnston and Narayanan, 2008; Spruston, 2008; Narayanan and Johnston, 2012), in the locus of plasticity in these channels (Frick et al., 2004; Narayanan and Johnston, 2007; Losonczy et al., 2008; Narayanan et al., 2010; Shah et al., 2010), and in calcium source localization and calcium propagation (Magee and Johnston, 1995; Augustine et al., 2003; Hertle and Yeckel, 2007; Ross, 2012) across the somatodendritic arbor. Consistent with this, and given our results with a single-compartmental model (necessitated by the tremendous computational complexity of calcium-dependent evolution), we postulate that the behavioral-state dependent intrinsic plasticity reported here would be dependent on the somatodendritic location of the neuronal compartment.

Testing this postulate would require development of specific experimental techniques and computational models to assess the impact of the self-regulating evolution of calcium homeostasis on changes in localization profiles of ion channels across the somatodendritic arbor. Experimental procedures would require direct measurement of somatodendritic channel properties under *in vivo* conditions, during different stages of sleep or behavior, with location-dependent afferent activity monitored in parallel (Agarwal et al., 2014; Schomburg et al., 2014). These experiments would provide direct answers to questions on whether dendritic channel localization profiles change as a function of activity switch (during specific stages of sleep or behavior), and if neurons implement an efficient form of neural coding that accounts the statistics of their afferent activity (Stemmler and Koch, 1999; Simoncelli and Olshausen, 2001; Simoncelli, 2003; Narayanan and Johnston, 2012).

Computational models, on the other hand, would have to explicitly account for somatodendritic differences in ion channel profiles, physiological measurements and calcium source localization (Berridge, 2006; Vacher et al., 2008; Kotaleski and Blackwell, 2010; Nusser, 2012; Ashhad and Narayanan, 2013; Hanus and Schuman, 2013; Rathour and Narayanan, 2014), apart from ensuring that spatial compartmentalization of the neuronal model is based on the calcium space constant rather than the electrical space constant (Koch and Zador, 1993; Zador and Koch, 1994; Ashhad and Narayanan, 2013). Second, these models would have to address the question on whether calcium homeostasis is maintained globally or locally, and ask if localization profiles of different channels were emergent properties consequent to the cell-autonomous calcium homeostasis process (Siegel et al., 1994; Rabinowitch and Segev, 2006, 2008). Third, consistent with the existence of several enzymes that act as calcium sensors (Liu et al., 1998), and the existence of several activity-dependent transcription factors (Dolmetsch, 2003), future models should extend beyond the single transcription factor-based analysis used in our model (see below). Finally, in our study, we have not accounted for neuromodulatory influences, and have resorted to a simplistic classification of afferent activity as theta versus SWR. Future experimental and computational studies on state-dependent calcium homeostasis should also account for differences in neuromodulatory activity during REM versus exploratory behavior and non-REM versus non-exploratory behavior (Vertes, 1984; Steriade, 2004; McCarley, 2007; Lee and Dan, 2012), and the impact of each of such differential neuromodulatory activity on ion channels and their plasticity (Fisher and Johnston, 1990; Hoffman and Johnston, 1999; Cantrell and Catterall, 2001; Marder and Thirumalai, 2002; Perez-Reyes, 2003; Biel et al., 2009; Marder, 2012; Marder et al., 2014).

What is the impact of such location-dependent plasticity in somato-dendritic channel properties on neuronal physiology and behaviorally relevant neural computation? First, such plasticity would alter the following physiological characteristics of these neuronal structures, each of which is known to exhibit location-dependence in a manner dependent on specific ion channel combinations: spectral selectivity (Narayanan and Johnston, 2007; Hu et al., 2009; Das and Narayanan, 2014), coincidence detection (Johnston et al., 2003; London and Häusser, 2005; Sjöström et al., 2008; Das and Narayanan, 2015), impedance phase (Narayanan and Johnston, 2008; Vaidya and Johnston, 2013), and supralinear summation (Losonczy and Magee, 2006; Losonczy et al., 2008; Sjöström et al., 2008; Spruston, 2008; Takahashi and Magee, 2009). Importantly, such state-dependent plasticity in channel conductances would not just reflect as changes in intrinsic neuronal physiology, but also express as changes in the amplitude and phase of local field potentials (LFP) and associated neuronal spike phases (Buzsáki et al., 2012; Schomburg et al., 2012; Einevoll et al., 2013; Reimann et al., 2013; Sinha and Narayanan, 2015). Future studies should test if such intrinsically-driven changes in LFP and spike phase could potentially form a cellular substrate for REM-shifting neurons (Mizuseki et al., 2011), a scenario where the phase shift is a consequence of intrinsic plasticity that occurred during an intervening switch to non-REM activity (Figs. 7–9, 11). Finally, extrapolating from recent studies that have demonstrated the importance of dendritic nonlinearities to place cell formation (Bittner et al., 2015; Sheffield and Dombeck, 2015), changes in dendritic sodium/calcium/potassium/HCN channels would alter the propensity for generating dendritic plateau potentials (Golding et al., 1999; Gasparini and Magee, 2002; Gasparini et al., 2004; Losonczy and Magee, 2006; Tsay et al., 2007; Losonczy et al., 2008), potentially resulting in changes in the place cell properties of the associated hippocampal neurons (Bittner et al., 2015).

### Implications for the assumption on a single transcription factor

In our model, we have assumed that the channel conductances are regulated by a single transcription factor (O'Leary et al., 2014), an assumption that significantly oversimplifies the complexities of neuronal transcription, where multiple transcription factors coexist (Bading et al., 1993; Dolmetsch, 2003; Lein et al., 2007; Alberini, 2009). This assumption implies that proportions of changes in channel conductances are correlated (Fig. 3*A*), resulting in correlated channel expression profiles (O'Leary et al., 2014). Although this assumption was motivated by correlated expression profiles of ion channels in certain neuronal subtypes (Toledo-Rodriguez et al., 2004; Schulz et al., 2006, 2007; Tobin et al., 2009; Amendola et al., 2012), detailed quantitative analysis of channel conductances and mRNA expression has not been performed in single hippocampal neurons. In the absence of such experimental data, not just at the cell body, but across the somatodendritic arbor (Hanus and Schuman, 2013; Rathour and Narayanan, 2014), model-based extrapolations about correlations in expression profiles of hippocampal channels or mRNAs would be incorrect, because the model outcome is just a direct consequence of the assumption involving a single transcription factor. Therefore, we restrict our inferences from this simple model to: (1) variable state-dependent plasticity of ionic conductances and intrinsic properties toward cell-autonomous maintenance of calcium homeostasis and (2) significant dissociation between different forms of homeostasis (see below), which also form testable predictions from our analysis. The question on whether the expression profiles of different channels/mRNAs are correlated or lack significant correlation in the presence of multiple transcription factors needs to be rigorously addressed both from experimental as well as theoretical standpoints.

Incorporation of multiple transcription factors into a model for cell-autonomous calcium homeostasis has been reported to result in unbounded production of mRNAs and channels (“windup”), leading to eventual loss of regulatory control (O'Leary et al., 2014). Although this constitutes a significant impediment to the incorporation of multiple transcription factors into models, this analysis was performed in a manner where the different transcription factors were independent of each other (O'Leary et al., 2014). Future theoretical studies should explore the possibility of avoiding such windup by coupling the multiple calcium sensors and multiple transcription factors through established signaling motifs, including negative feedback mechanisms (Thattai and van Oudenaarden, 2001; Losick and Desplan, 2008; Yu et al., 2008; Kotaleski and Blackwell, 2010; Cheong et al., 2011). Experimental studies should explore the relationships between the different transcription factors, mRNAs and channel conductances across the somatodendritic arbor of single hippocampal neurons (Dolmetsch, 2003; Hanus and Schuman, 2013).

### Dissociations between different forms of homeostasis

It is clear from our analyses here, and from several others in the literature, that there are significant dissociations between different forms of homeostasis. First, homeostasis in functional properties, including synaptic plasticity profiles (Anirudhan and Narayanan, 2015), could emerge with disparate conductance values for the constituent ion channels and synaptic conductances (Golowasch and Marder, 1992; Golowasch et al., 2002; MacLean et al., 2005; Schulz et al., 2006, 2007; Taylor et al., 2009; Rathour and Narayanan, 2012a, 2014), suggesting that homeostatic maintenance of single channels at specific conductance values is not essential for maintaining functional or plasticity profile homeostasis. Second, for maintenance of calcium homeostasis across neurons in a network (O'Leary et al., 2013), or in a neuron that receives state-dependent switch in afferent activity (Figs. 3–11), it is not essential that functional homeostasis across different measurements is maintained. Specifically, despite maintenance of calcium homeostasis across models receiving identical temporal evolution of afferent activity, we noted that the conductance values (Figs. 3, 8) and physiological measurements (Figs. 3–6, 9–11) were significantly variable across these models, with some models manifesting measurements beyond what is expected from CA1 pyramidal neurons. Additionally, a significant proportion of neurons did not restore their intrinsic properties despite restoration of specific type of activity after an intervening switch to a different type of activity (Figs. 9–11). Finally, although calcium homeostasis was achieved across all neuronal models, there was significant variability (across models) in the firing rate and in pattern of firing in response to the same afferent activity (Figs. 3,7; O'Leary et al., 2013, 2014). Together, these results clearly establish that maintenance of calcium homeostasis neither translates to nor follows from any of channel/functional/firing-rate forms of homeostasis, outlining critical dissociations between these forms of homeostasis.

Future experiments should therefore explore the specific form of homeostasis maintained by individual neurons and their dendritic arbor when subjected to behavioral state-dependent afferent activity during different stages of sleep and behavior. Do channel properties/localization and intrinsic functional properties change in the process of maintaining calcium homeostasis? Or, do neurons implement a mechanism where all forms of homeostasis—including that in spatially distributed channel properties, intrinsic response properties, plasticity profiles, and calcium levels—are concurrently maintained across all somatodendritic locations of the neuron, with firing rate homeostasis emerging as an overall consequence? Should a homeostatic mechanism account not just for the average calcium level in a neuron, but also make provisions for the homeostasis in input-output profiles, intrinsic response properties and synaptic/intrinsic plasticity profiles of the neuron through synergistic interactions between synaptic and intrinsic neuronal properties (LeMasson et al., 1993; Liu et al., 1998; Triesch, 2007; Turrigiano, 2011; Honnuraiah and Narayanan, 2013; O'Leary et al., 2014; Anirudhan and Narayanan, 2015)? Finally, whereas homeostasis covers only one aspect of neuronal function, the other core function (especially of hippocampal neurons), is encoding of new information. Juxtaposed against questions on various forms of homeostasis is the fundamental issue of how neurons change their properties toward encoding new information, without jeopardizing any or some forms of homeostasis. Therefore, further exploration into behavioral state-dependent evolution of homeostasis should account for encoding as a critical aspect of neuronal function that depends on changes in intrinsic and/or synaptic properties (Narayanan and Johnston, 2012), apart from exploring the relationships between different forms of homeostasis.

## Synthesis

The decision was a result of the Reviewing Editor Douglas Bayliss and the peer reviewers coming together and discussing their recommendations until a consensus was reached. A fact-based synthesis statement explaining their decision and outlining what is needed to prepare a revision is listed below. The following reviewers agreed to reveal their identity: Astrid Prinz, Ronald Harris-Warrick

Synthesis

The reviewers agree that this work provides new understanding with respect to homeostatic compensation mechanisms, specifically by demonstrating that calcium homeostasis is not necessarily sufficient for functional/physiological homeostasis. As detailed in the comments provided, there are a number of points of presentation and interpretation that should be clarified. Many issues were shared between the reviewers. For example, some explanation is needed for why all the currents were modulated by a single transcription factor. Also, both reviewers found that the discussion could be extended at multiple points to provide more "big-picture" meaning to the results, and to examine more deeply the consequences of the assumptions in the model for the overall conclusions. Other specific points can be found in their comments (below).

Reviewer 1

Major:

A major result of the presented work is that cell-autonomous calcium homeostasis is not sufficient for functional homeostasis. This is in contrast to some earlier models that assumed functional homeostasis on the basis of calcium homeostasis. If calcium homeostasis does not maintain functional cell output, then what is it good for? Why does the cell apparently work so hard to maintain calcium concentration? We would appreciate some comments on this question in the discussion section.

On a related note, the authors use two different input patterns onto hippocampal CA1 pyramidal neurons and argue that these inputs mimic hippocampal activity in different stages of sleep. However, after showing that the modeled calcium homeostasis does not support functional homeostasis for either input pattern, the authors fail to relate this finding back to the role of sleep and the different oscillation patterns that occur in different sleep or activity stages, and to the existing literature they cite in the introduction. Again, an addition to the discussion to further explain the big-picture meaning of the results would be appreciated.

For average reader, who may not be an expert on hippocampal dynamics, it would be helpful if the authors could describe, or better yet, show in a figure, what the typical voltage and calcium response of hippocampal pyramidal neurons to either of the two tested input patterns looks like in vivo, and how well their hand-tuned base model neuron matches that. This would make it easier to evaluate how functional or pathological some of the simulated behaviors really are, for example those in Figure 3B or Figure 7.

Regarding different classes of neurons, and different types of plasticity and robustness, for example as shown in Figure 7: the statement that there are two classes of model neurons in play here, plastic and robust, is over-simplified. For example, Figures 7 D and F both show neurons that are plastic in terms of their maximal conductances, but 7F returns to its initial activity pattern and could thus be called functionally robust, whereas 7D is not functionally robust. Because the distinction between different forms of homeostasis (calcium vs. intrinsic properties vs. functional) is an important point of this paper, these distinctions should be made more carefully.

We would like to see some more discussion and motivation for the fact that the authors chose a homeostasis model that corresponds to just one transcription factor regulating all conductances (see line 488), which leads to the maximal conductance values all scaling together. What is known about the transcription factors controlling ion channels in hippocampal neurons? In STG neurons, which the authors use as a motivation, conductances can show pairwise or higher correlations, but are certainly not all correlated to each other.

Minor:

Terminology: We were somewhat confused by the terminology used by the authors in multiple places in the manuscript, including the title. The paper establishes models of calcium homeostasis and simulates the time course of calcium concentration, but the homeostatic mechanism itself stays constant over time. So why talk about "regulation of calcium homeostasis" (title) or "maintenance of calcium homeostasis" (first line of abstract)? That seems redundant. Similar redundant terminology occurs throughout the paper.

Calcium diffusion (page 8, top): the paper states that radial diffusion of calcium was modeled by compartmentalizing the cylindrical model into four annuli, implying that the calcium concentration will be different in different annuli. Which of the four concentrations was used in the rest of the model to represent cytosolic calcium? We do not understand how the compartmentalization of calcium relates to the use of a single calcium value in the rest of the equations. Please explain in more detail.

We suggest that the lower and upper bounds on output measures that constrained the original model population (see Table 2) be shown in Figures 3 - 11 where appropriate so that the reader can judge whether and how far the homeostatic regulation took the various models out of the functional range of measures.

Histograms in Figure 4 and onward: the histograms are shown as lines with the area underneath the line filled, which leads to the front histograms occluding the back ones. We suggest showing the histograms as colored lines only, so that the reader can better see what is going on.

Related to that, the text states that (for example in Figure 5) "no noticeable patterns emerged from the histogram". Some panels, for example 5E, appear to be showing systematic trends. How did the authors judge whether there is a pattern or not? Judgement by eye rather than statistically is ok, we would just like to know how it was done.

Figure 2: the large triangle of tiny plots with 78 dots in each is fairly useless, because even when zooming in, it is practically impossible to see what is going on in any individual little plot. We suggest simplifying the figure by showing parts B and C and zoomed-in versions of 4 or 5 individual plots, preferably the red and blue examples and a few examples with weak or no correlations.

There are several instances of the phrase "against this", for example in line 464 and line 575. This is not a common use of the term "against". Please rephrase.

Some of the figure captions are a bit repetitive. For example, the experimental switch protocol already used in Figures 7 and 8 does not need to again be described in great detail in the caption to Figure 9.

The authors suggest that future studies examine the role of neuronal intrinsic properties (as opposed to synaptic properties) towards activity homeostasis. Studies exist in hippocampal neurons and other systems (STG etc.) that already do this, for example, Honnuraiah and Narayanan, 2013, PLoS One. Such studies should be cited.

Line 37: "switch" should be "switches"

Line 128: "current were modeled as combination" should be "current was modeled as a combination"

Line 420: delete "number of"

Reviewer 2

This is a very interesting modeling study that tests whether maintenance of calcium homeostasis is the critical parameter for neuronal functional homeostasis. A series of detailed models of hippocampal pyramidal neurons were made, with 12 conductances, each of which could have variable initial parameters in maximal conductance and kinetics. 78 of these models (2% of the total tested) showed baseline activity within the normal range of pyramidal neurons based on seven activity parameters. When driven with different kinds of synaptic drive and driven to return to calcium homeostasis, the models changed their firing rates in vary diverse ways. Further, the pattern of change depended on the order of changes in synaptic drive; many neurons did not return to their original firing patterns when returned from a second pattern to the original first pattern. This suggests that plasticity based on calcium homeostasis by itself cannot assure physiological homeostasis, and that the varying components of homeostasis (intrinsic, synaptic, genetic, calcium-dependent) may interact in complex ways to assure neuronal homeostasis.

This conclusion depends on several assumptions of the model.

First and foremost, as pointed out in the discussion, the model couples the changes of all the currents in parallel by driving their RNA transcription under control of a single calcium-dependent transcription factor. This is very unlikely to be true, based on studies in simpler systems. If the currents could increase or decrease more independently of one another (though some may show correlations, as in the stomatogastric ganglion), or if multiple transcription factors had different calcium sensitivity, these results may change substantially. If so, it would be interesting to see whether the weak correlations in parameters in Fig. 2 are maintained or change as the state of the system changes.

Second, the model assumes that the synaptic activity pattern is constant long enough for changes in gene expression and channel synthesis and insertion to reach a new steady state. This is probably not correct either, as the pattern can change rapidly during sleep states. The relevant rate constants for channel insertion are not well understood, but probably much slower. They will vary depending on whether the regulation occurs locally (for example, in dendrites with local protein synthesis) or only somatically, and, based on other studies, are likely to be very different for different channels. Thus, a model linking channel density or properties to calcium levels may only be relevant for much slower changes than the rapid synaptic changes modeled here.

Finally, due to the constraints of the computational time for these very complex simulations, the model is based on a single compartment, suggesting that a single rule regulates all parts of the neuron.

The authors discuss all these points, yet do not discuss the consequences of them for their conclusions.

Minor points:

1) p. 20, line 433 and Fig 5A: are the mV values in Fig 5A accurate, so that at 400 nm/s input the neuron is oscillating over a 250 mV range? How can any action potentials arise at such positive values?

2) Fig 5A: how were the "typical" records selected from the 78 model results, and how typical are they? For example, the neuron driven by 400 nm/s has very small action potentials, yet the majority of neurons in the histogram in Fig. 5C have 90 mV action potentials under this drive. Please explain.

3) p. 22, line 470ff: give an estimate at this point of the percentages of robust vs. plastic neurons.

REPLIES TO REVIEWERS' COMMENTS

We thank the reviewers for their constructive comments and for their positive response to our manuscript. In response to the reviewers' positive comments, we have made significant changes to the manuscript, specifically addressing the reviewers' and reviewing editors' concerns. We believe that these changes overall have significantly strengthened the manuscript, especially allowing us to explore the implications for our conclusions to neuronal physiology. Grateful thanks to the reviewers and to the reviewing editor!

REPLY TO THE SYNTHESIS OF REVIEWS

Review: The reviewers agree that this work provides new understanding with respect to homeostatic compensation mechanisms, specifically by demonstrating that calcium homeostasis is not necessarily sufficient for functional/physiological homeostasis. As detailed in the comments provided, there are a number of points of presentation and interpretation that should be clarified. Many issues were shared between the reviewers. For example, some explanation is needed for why all the currents were modulated by a single transcription factor. Also, both reviewers found that the discussion could be extended at multiple points to provide more "big-picture" meaning to the results, and to examine more deeply the consequences of the assumptions in the model for the overall conclusions. Other specific points can be found in their comments (below).

Reply: We thank the reviewers for their positive response. Please note that we have significantly expanded the discussion section to present detailed analysis with reference to each of the points raised by the reviewers. With reference to the major concern by both the reviewers about the assumption on the use of a single transcription factor to regulate all conductances, we have now added a new subsection to the discussion section ("Implications for the assumption on single transcription factor") where this assumption is explored in detail. Additionally, with reference to the "big picture" meaning of the results, we have expanded the two discussion subsections on "Behavioral state-dependent changes in neuronal intrinsic properties" and "Would behavioral state-dependent intrinsic plasticity in a neuronal compartment be dependent on its somatodendritic location?". In these subsections, we have explored in detail the implications for our results on several aspects of neuronal physiology, starting from single-cell properties to behaviorally relevant neural computation. The specific changes made in the manuscript are detailed below in the point-by-point replies to the reviewers' comments.

-

REPLIES TO REVIEWER 1

1. A major result of the presented work is that cell-autonomous calcium homeostasis is not sufficient for functional homeostasis. This is in contrast to some earlier models that assumed functional homeostasis on the basis of calcium homeostasis. If calcium homeostasis does not maintain functional cell output, then what is it good for? Why does the cell apparently work so hard to maintain calcium concentration? We would appreciate some comments on this question in the discussion section.

Reply: The question on what constitutes homeostasis is still open. Although several postulates exist about whether this should be firing rate or calcium levels or overall synaptic weights or overall conductance levels of different channels or specific physiological phenotype of the cell, the answer to the question on which form of homeostasis the cell "chooses" to maintain is still open. Given this, our results, in conjunction with several previous observations, clearly demonstrate the significant dissociation between different forms of homeostasis. Our results also suggest that maintenance of calcium homeostasis alone is not sufficient towards maintenance of other forms of homeostasis, and therefore the postulates on which specific form of homeostasis is important needs revisiting and reanalysis.

Given this dissociation, we have incorporated suggestions, in the discussion section, for future experiments to explore the specific form of homeostasis maintained by individual neurons and their dendritic arbor when subjected to behavioral state-dependent afferent activity. We have incorporated questions about whether channel properties/localization and intrinsic functional properties change in the process maintaining calcium homeostasis? Or, if neurons implement an alternate mechanism where all forms of homeostasis, - including that in spatially distributed channel properties, intrinsic response properties, plasticity profiles and calcium levels, - are concurrently maintained across all somatodendritic locations of the neuron, with firing rate homeostasis emerging as an overall consequence? We have also discussion points asking if a homeostatic mechanism account not just for the average calcium level in a neuron, but also make provisions for the homeostasis in input-output profiles, intrinsic response properties and synaptic/intrinsic plasticity profiles of the neuron?

These points are mentioned in the discussion section (Discussion section, subsection on "Dissociations between different forms of homeostasis").

2. On a related note, the authors use two different input patterns onto hippocampal CA1 pyramidal neurons and argue that these inputs mimic hippocampal activity in different stages of sleep. However, after showing that the modeled calcium homeostasis does not support functional homeostasis for either input pattern, the authors fail to relate this finding back to the role of sleep and the different oscillation patterns that occur in different sleep or activity stages, and to the existing literature they cite in the introduction. Again, an addition to the discussion to further explain the big-picture meaning of the results would be appreciated.

Reply: We thank the reviewer for raising this point. In response to the reviewer's suggestion, we have now (i) significantly expanded the discussion subsection on "Behavioral state-dependent changes in neuronal intrinsic properties" to suggest that memory consolidation during changes in afferent rhythms should also account for intrinsic plasticity as a putative cellular substrate. Here we revisit the references cited in the introduction, and explore the implications for our results to observations presented in those articles; (ii) suggested specific experiments to the discussion subsection on "Would behavioral state-dependent intrinsic plasticity in a neuronal compartment be dependent on its somatodendritic location?" to measure activity patterns (extracellular and intracellular) during different stages of sleep and behavior to assess state-dependent intrinsic changes. This subsection has been significantly expanded to elucidate the implications for location-dependent regulation of intrinsic properties on several aspects of neuronal physiology, starting from single-cell properties to behaviorally-relevant neural computation; and (iii) explored the implications for the dissociation between functional and calcium homeostasis from the standpoint of switches in afferent activity patterns in the discussion subsection on "Dissociations between different forms of homeostasis".

3. For average reader, who may not be an expert on hippocampal dynamics, it would be helpful if the authors could describe, or better yet, show in a figure, what the typical voltage and calcium response of hippocampal pyramidal neurons to either of the two tested input patterns looks like in vivo, and how well their hand-tuned base model neuron matches that. This would make it easier to evaluate how functional or pathological some of the simulated behaviors really are, for example those in Figure 3B or Figure 7.

Reply: Please note that the waveforms, their frequencies (both theta and ripples), and their rate of occurrence (for ripples) were taken from extracellular LFPs (constitutive of afferent inputs) and intracellular experimental data. Consequently, we model theta-frequency inputs as pure sinusoids of 8 Hz, and the SWR complex using this formulation:

where t is time in ms, fripple is the ripple frequency set at 150 Hz. Each SWR waveform lasted for 150 ms and repeat at a frequency of 3 Hz. These explanations are now provided as part of the Methods section, under the subsection on "Assessing state-dependent evolution of calcium homeostasis", including the equation above, with appropriate references. Representative conductance plots and voltage responses of the model to these theta and SWR afferent inputs are shown in Fig. 6, with the equation referred in the legend of Fig. 6.

4. Regarding different classes of neurons, and different types of plasticity and robustness, for example as shown in Figure 7: the statement that there are two classes of model neurons in play here, plastic and robust, is over-simplified. For example, Figures 7 D and F both show neurons that are plastic in terms of their maximal conductances, but 7F returns to its initial activity pattern and could thus be called functionally robust, whereas 7D is not functionally robust. Because the distinction between different forms of homeostasis (calcium vs. intrinsic properties vs. functional) is an important point of this paper, these distinctions should be made more carefully.

Reply: We agree that it is oversimplified, and is more a continuum of conductance plasticity than two distinct classes (as seen from the histograms in Figs. 8 and 11). We have emphasized this when we expand on Fig. 7 in the Results section. Additionally, we have also mentioned the distinction between changes in activity patterns observed during the presentation of activity pattern, and changes in ion channel properties. We regret the ambiguity, and thank the reviewer for providing us the opportunity to rectify this. We have made multiple changes to the results subsection on "Neuronal conductances exhibited differential robustness to an intervening switch in the type of afferent activity" where we have rectified this ambiguity, and have brought this distinction into the overall theme on dissociations between different forms of homeostasis.

5. We would like to see some more discussion and motivation for the fact that the authors chose a homeostasis model that corresponds to just one transcription factor regulating all conductances (see line 488), which leads to the maximal conductance values all scaling together. What is known about the transcription factors controlling ion channels in hippocampal neurons? In STG neurons, which the authors use as a motivation, conductances can show pairwise or higher correlations, but are certainly not all correlated to each other.

Reply: In response to the reviewer's suggestion, we have now incorporated a new discussion subsection that explores this assumption in detail. Please refer to the discussion subsection "Implications for the assumption on single transcription factor". We have mentioned clearly that this assumption constitutes a significant oversimplification, and have suggested alternate routes to build models with multiple transcription factors.

6. Terminology: We were somewhat confused by the terminology used by the authors in multiple places in the manuscript, including the title. The paper establishes models of calcium homeostasis and simulates the time course of calcium concentration, but the homeostatic mechanism itself stays constant over time. So why talk about "regulation of calcium homeostasis" (title) or "maintenance of calcium homeostasis" (first line of abstract)? That seems redundant. Similar redundant terminology occurs throughout the paper.

Reply: Please note that calcium homeostasis in the model is actively regulated by plasticity in ionic conductances. Specifically, changes in calcium concentration are inevitable with changes in afferent activity. To maintain this calcium concentration at a target level (calcium homeostasis), our model employs a cell-autonomous self-regulatory mechanism that adjusts ionic conductances towards achieving that goal.

7. Calcium diffusion (page 8, top): the paper states that radial diffusion of calcium was modeled by compartmentalizing the cylindrical model into four annuli, implying that the calcium concentration will be different in different annuli. Which of the four concentrations was used in the rest of the model to represent cytosolic calcium? We do not understand how the compartmentalization of calcium relates to the use of a single calcium value in the rest of the equations. Please explain in more detail.

Reply: Please note that cytosolic calcium was represented by the calcium concentration in the outermost annulus (Carnevale and Hines, The Neuron Book, 2006). This is to account for the calcium on and off mechanisms on the plasma membrane and for the sharp decay of calcium with distance with a decay tau on the order of 0.1 Âµm (Zador and Koch, J. Neuroscience, 1994). These points are now mentioned in the Methods section where the annuli are described (subsection on "Calcium dynamics").

8. We suggest that the lower and upper bounds on output measures that constrained the original model population (see Table 2) be shown in Figures 3 - 11 where appropriate so that the reader can judge whether and how far the homeostatic regulation took the various models out of the functional range of measures.

Reply: Please note that we have now incorporated these bounds in figures 4-6 as per the suggestion. In other figures, they were either not necessary (Fig. 7-8, 11) or their incorporation made the figure look cluttered (Fig. 3, 9-10). We thank the reviewer for the suggestion.

9. Histograms in Figure 4 and onward: the histograms are shown as lines with the area underneath the line filled, which leads to the front histograms occluding the back ones. We suggest showing the histograms as colored lines only, so that the reader can better see what is going on.

Reply: Please note that we tried making them as colored lines as opposed to having them filled, but found that to be more cluttered. Therefore we respectfully request that we be allowed to retain this as it is.

10. Related to that, the text states that (for example in Figure 5) "no noticeable patterns emerged from the histogram". Some panels, for example 5E, appear to be showing systematic trends. How did the authors judge whether there is a pattern or not? Judgement by eye rather than statistically is ok, we would just like to know how it was done.

Reply: Please note that this was just a qualitative observation based on the shapes of the histogram. To emphasize this, we have modified the statement from "no noticeable patterns emerged from the histogram" to "with no qualitative differences observed in the measurement variability".

11. Figure 2: the large triangle of tiny plots with 78 dots in each is fairly useless, because even when zooming in, it is practically impossible to see what is going on in any individual little plot. We suggest simplifying the figure by showing parts B and C and zoomed-in versions of 4 or 5 individual plots, preferably the red and blue examples and a few examples with weak or no correlations.

Reply: The plots in Figure 2 serve multiple purposes. First, the histograms depicted on the bottom-most panels demonstrate that each model parameter spanned the entire minimum-maximum stretch. A restricted span for any of these model parameters would imply that the parameter is a critical one, and constitutes a significant constriction in the valid model parametric space. Second, the individual scatter plots also show that the valid models spanned the entire stretch, and not clusters for each pairwise parametric combination. Studies that involve global sensitivity analysis have shown these plots to emphasize these two points (e.g., Taylor et al., 2009, Rathour and Narayanan, 2012, 2014). As the emphasis is not towards details of individual plots, but is on the overall scatter associated with each pairwise combination, we respectfully request the reviewer to allow us to retain the plots as they are. Thank you.

12. There are several instances of the phrase "against this", for example in line 464 and line 575. This is not a common use of the term "against". Please rephrase.

Reply: "Against this" has been replaced by "In this context" at both the mentioned locations. We thank the reviewer for point this to us.

13. Some of the figure captions are a bit repetitive. For example, the experimental switch protocol already used in Figures 7 and 8 does not need to again be described in great detail in the caption to Figure 9.

Reply: Please note that the switch protocol has now been deleted from the captions to Figs. 9-10. Thank you.

14. The authors suggest that future studies examine the role of neuronal intrinsic properties (as opposed to synaptic properties) towards activity homeostasis. Studies exist in hippocampal neurons and other systems (STG etc.) that already do this, for example, Honnuraiah and Narayanan, 2013, PLoS One. Such studies should be cited.

Reply: Please note that the reference suggested by the reviewer, and other references from the STG system have now been appropriately cited at the suggested location and others. Thank you.

15. Line 37: "switch" should be "switches"

Reply: Please note that the line now reads with "switches" instead of "switch". Thank you.

16. Line 128: "current were modeled as combination" should be "current was modeled as a combination"

Reply: Please note that the grammatical slip has now been corrected. Thank you.

17. Line 420: delete "number of"

Reply: Please note that the phrase has now been deleted. Thank you.

-

REPLIES TO REVIEWER 2

1. First and foremost, as pointed out in the discussion, the model couples the changes of all the currents in parallel by driving their RNA transcription under control of a single calcium-dependent transcription factor. This is very unlikely to be true, based on studies in simpler systems. If the currents could increase or decrease more independently of one another (though some may show correlations, as in the stomatogastric ganglion), or if multiple transcription factors had different calcium sensitivity, these results may change substantially. If so, it would be interesting to see whether the weak correlations in parameters in Fig. 2 are maintained or change as the state of the system changes.

Reply: In response to the reviewer's suggestion, we have now incorporated a new discussion subsection that explores this assumption in detail. Please refer to the discussion subsection "Implications for the assumption on single transcription factor". We have mentioned clearly that this assumption constitutes a significant oversimplification, and have suggested alternate routes to build models with multiple transcription factors.

2. Second, the model assumes that the synaptic activity pattern is constant long enough for changes in gene expression and channel synthesis and insertion to reach a new steady state. This is probably not correct either, as the pattern can change rapidly during sleep states. The relevant rate constants for channel insertion are not well understood, but probably much slower. They will vary depending on whether the regulation occurs locally (for example, in dendrites with local protein synthesis) or only somatically, and, based on other studies, are likely to be very different for different channels. Thus, a model linking channel density or properties to calcium levels may only be relevant for much slower changes than the rapid synaptic changes modeled here.

Reply: We agree with the reviewer on all three counts and have made appropriate mentions in the manuscript:

(a) A constant synaptic activity pattern is physiologically infeasible, although it is well established that the propensity of different activity patterns (in terms of frequency bands, for instance) is higher during specific behavioral states. This is now presented in the manuscript in the discussion subsection on "Behavioral state-dependent changes in neuronal intrinsic properties".

(b) Although several in vitro experiments have shown plasticity in several ion channels to occur in a matter of minutes (e.g., Shah et al., Trends in Neurosciences, 2011 and references therein) and have demonstrated that changes in ion channels and intrinsic properties can be concurrent with synaptic changes (e.g., Frick et al., Nature Neuroscience, 2004; Fan et al., Nature Neuroscience, 2005; Kim et al., Neuron, 2007; Narayanan and Johnston, Neuron, 2007; Losonczy et al., Nature, 2008; Lin et al., Nature Neuroscience, 2008; Narayanan et al., Neuron, 2010), the time constants for channel plasticity under in vivo conditions and their relationship to the extent of synaptic activity patterns need further exploration. This is now presented in the manuscript in the discussion subsection on "Behavioral state-dependent changes in neuronal intrinsic properties".

(c) The somatodendritic location-dependence of regulatory mechanisms needs to be explored in detail, considering different channels individually and collectively (Hanus and Schuman, Nature Reviews Neuroscience, 2013; Rathour and Narayanan, Proc. Nat. Acad. Sci. (USA), 2014). This is now presented in the manuscript in the discussion subsection on "Would behavioral state-dependent intrinsic plasticity in a neuronal compartment be dependent on its somatodendritic location?".

We thank the reviewer for raising these issues and providing us the opportunity to explore the implications for our assumptions in detail.

3. Finally, due to the constraints of the computational time for these very complex simulations, the model is based on a single compartment, suggesting that a single rule regulates all parts of the neuron.

Reply: Please note that we have explored this simplification in considerable detail in the discussion subsection on "Would behavioral state-dependent intrinsic plasticity in a neuronal compartment be dependent on its somatodendritic location?". As the reviewer rightly points out, given the complexity associated with reaction-diffusion systems that encompasses the entire morphology, and given the large simulation running time for these kinds of models, it was rather infeasible to account for morphological complexity as well. Therefore we had to resort to this simple single compartmental model where a single-compartmental model is studied. This model was sufficient to underscore our key observations on (a) variable state-dependent plasticity of ionic conductances and intrinsic properties towards cell-autonomous maintenance of calcium homeostasis and (b) significant dissociation between different forms of homeostasis. However, as is clear from the discussion subsection, we are not extrapolating this to mean that a single rule regulates all parts of the neuron. Please note that this is also clearly stated as a postulate in the discussion subsection, where we state: "...we postulate that the behavioral-state dependent intrinsic plasticity reported here would be dependent on the somatodendritic location of the neuronal compartment.". Please note that we have also significantly expanded the aforementioned discussion section to account for implications for location-dependent regulation of intrinsic properties.

4. p. 20, line 433 and Fig 5A: are the mV values in Fig 5A accurate, so that at 400 nm/s input the neuron is oscillating over a 250 mV range? How can any action potentials arise at such positive values?

Reply: Please note that although the membrane potential oscillated over a large range when the sinusoidal amplitude was set to extremely high values. These oscillations were on the hyperpolarizing direction (where the driving force for the AMPAR/NMDAR conductances were large). On the depolarizing side, an action potential was elicited once the membrane potential crossed threshold, and the amplitude of the action potential did not cross the sodium reversal potential of +55 mV. This is now stated in the legend of Fig. 5.

5. Fig 5A: how were the "typical" records selected from the 78 model results, and how typical are they? For example, the neuron driven by 400 nm/s has very small action potentials, yet the majority of neurons in the histogram in Fig. 5C have 90 mV action potentials under this drive. Please explain.

Reply: Please note that the action potential (AP) amplitude used to characterize the neuron is not the amplitude of the action potential in the neuron's response to the afferent activity (shown in Fig. 5A). Rather, as we have mentioned in the Methods section, AP amplitude was calculated as the difference between the peak voltage of the last action potential and the resting membrane voltage (-65 mV) when a 250 pA current was injected into the neuron for 500 ms. Please also note that the action potentials in Fig 5A, if measured from -65 mV would have similar amplitudes. They appear small because of the large membrane oscillations of 250 mV that span the hyperpolarized direction. We regret the ambiguity, and thank the reviewer for providing us the opportunity to rectify this. We have now clarified this in the legend for Fig. 5.

6. p. 22, line 470ff: give an estimate at this point of the percentages of robust vs. plastic neurons.

Reply: The percentages (robust: ∼20%; plastic: ∼80%) are now stated as part of the sentence mentioned by the reviewer. Thank you.
